# Adaptive Bayesian Learning and Forecasting of Epidemic Evolution–Data Analysis of the COVID-19 Outbreak

**DOI:** 10.1109/ACCESS.2020.3019922

**Published:** 2020-09-30

**Authors:** DOMENICO GAGLIONE, PAOLO BRACA, LEONARDO MARIA MILLEFIORI, GIOVANNI SOLDI, NICOLA FORTI, STEFANO MARANO, PETER K. WILLETT, KRISHNA R. PATTIPATI

**Affiliations:** 1 NATO STO Centre for Maritime Research and Experimentation (CMRE), 19126 La Spezia, Italy; 2 Dipartimento di Ingegneria dell’Informazione ed Elettrica e Matematica Applicata (DIEM), University of Salerno, 84084 Fisciano, Italy; 3 Department of Electrical and Computer Engineering, University of Connecticut, Storrs, CT 06269-4157, USA

**Keywords:** SARS-CoV-2, Bayesian sequential estimation, ensemble forecasting, compartmental model, pandemic tracking, pandemic prediction

## Abstract

Since the beginning of 2020, the outbreak of a new strain of Coronavirus has caused hundreds of thousands of deaths and put under heavy pressure the world’s most advanced healthcare systems. In order to slow down the spread of the disease, known as COVID-19, and reduce the stress on healthcare structures and intensive care units, many governments have taken drastic and unprecedented measures, such as closure of schools, shops and entire industries, and enforced drastic social distancing regulations, including local and national lockdowns. To effectively address such pandemics in a systematic and informed manner in the future, it is of fundamental importance to develop mathematical models and algorithms to predict the evolution of the spread of the disease to support policy and decision making at the governmental level. There is a strong literature describing the application of Bayesian sequential and adaptive dynamic estimation to surveillance (tracking and prediction) of objects such as missiles and ships; and in this article, we transfer some of its key lessons to epidemiology. We show that we can reliably estimate and forecast the evolution of the infections from daily — and possibly uncertain — publicly available information provided by authorities, e.g., daily numbers of infected and recovered individuals. The proposed method is able to estimate infection and recovery parameters, and to track and predict the epidemiological curve with good accuracy when applied to real data from Lombardia region in Italy, and from the USA. In these scenarios, the mean absolute percentage error computed after the lockdown is on average below 5% when the forecast is at 7 days, and below 10% when the forecast horizon is 14 days.

## I. INTRODUCTION

### A. MOTIVATION AND BACKGROUND

Beginning in early December 2019, Chinese health authorities have been detecting and monitoring an increasing number of pneumonia cases in the city of Wuhan, a province of Hubei. The pneumonia, later named COVID-19, is caused by a new strain of Coronavirus, and is technically referred to as the severe acute respiratory syndrome Coronavirus 2 (SARS-CoV-2) [1]. As of August 23, 2020, more than 23 million people worldwide have been infected, and over 800 thousand have died. In March 2020, a series of events have pushed many governments to take extraordinary social measures. These events include the lack of effective cures and vaccines, the exponentially increasing number of individuals requiring recovery in intensive care units, and the announcement by the World Health Organization (WHO) of a Coronavirus pandemic on March 11, 2020 [2]. The adopted measures included closure of schools, universities, shops, industries, public and cultural places, the prohibition of mass gatherings, travel bans, and extreme social distancing, including local and national lockdowns. The main aim of these measures is to slow down the infection rate and alleviate the pressure on healthcare systems, in order to ensure care to all individuals stricken by the virus. Indeed, after the adoption of these measures, most countries have seen a decrease in the daily numbers of infected individuals. In order to prevent another exponential rise in infections as the restrictive measures are progressively relaxed, it is of crucial importance to develop mathematical models and algorithms to track and forecast the evolution of the infection with acceptable accuracy, which can help authorities to make informed and timely decisions. Improving our ability to model and forecast is also of paramount importance to better address future pandemic outbreaks [3].

The algorithm proposed in this article builds on the concept of compartmental epidemiological models, which assume that a given population is divided into a fixed number of compartments. Each compartment represents an epidemic state that an individual can occupy. The flow dynamics from one compartment to another are modeled as a set of stochastic differential equations that we discretize according to the discrete nature of the available data, i.e., daily update on the number of infected, recovered, dead, etc. In the standard SIR model, proposed in the pioneering study on mathematical theory of epidemics by Kermack and McKendrick [4], it is assumed that the entire population, e.g., of a city, a region, or a nation, is constant and divided into three compartments (population subgroups), namely, susceptible (S), infected (I), and recovered (R) individuals. Moreover, it is assumed that an infected individual infects a susceptible one at a given rate *β* [5]. Once infected, the individual is removed from the compartment of susceptibles and enters the infected compartment. Each infected person runs through the course of the disease, and eventually is removed from the number of those who are still infected either by recovery or death, thus exiting the system at “recovery” rate *γ*; the recovered people are considered permanently immune.^1^ The ratio *β*/*γ* is called the contact ratio, and represents the mean number of people the infected individual comes in contact with.

The SIR model is simple, yet very successful and useful in practice. Over the years, several more sophisticated extensions have been proposed to account for more compartments and other salient aspects of the epidemics. For example, a person who comes in contact with an infected individual and contracts the infection might not develop the symptoms immediately but only with a certain delay, called incubation period; in the case of COVID-19, this delay is around 3-15 days with a median of 5.2 days [6]. The SEIR model accounts for this circumstance by adding a further compartment that represents exposed — but not yet contagious — people [7], [8]. That is, susceptible individuals who contract the virus, pass to the exposed compartment (E) before evident symptoms appear and the person is confirmed as infected. The SEIRQ model is a further extension that also accounts for quarantined people [9]. Restriction measures are directly taken into account in the recently proposed SIR-X model [10] that, introducing an additional mechanism, removes susceptibles from the transmission process when the measures become effective.

A critical epidemiological characteristic for the pandemic potential of an emergent respiratory virus is represented by the undocumented, but infectious, cases. In contrast with the documented infectious cases, they often experience mild, limited, or no symptoms at all, and therefore, since they are generally not tested, remain undetected. These are the so-called asymptomatic cases in the context of COVID-19 pandemic. Based on their contagiousness and numbers, they can expose a far greater portion of the population to the virus than would otherwise occur. Li *et al.* [11] present a model-inference framework to estimate the contagiousness and proportion of undocumented infections in China before and after the lockdown in Wuhan.

Most of the compartmental models described so far consider the disease spread inside a unique and single population: a city, a region, a nation. In contrast, metapopulation models add a further spatial dimension, by interpreting the population as a network of multiple spatially separated subpopulations (nodes), e.g., multiple cities in the same region; the connections from one subpopulation to another are represented by movements (“diffusion”) of persons. Such interconnections represent contacts such as commuting to work, second homes, or national and international travels. In such a scenario, the diffusion of the infection is not only caused by the contacts among susceptibles and infectious people within each subpopulation, but also by the spatial interactions among the different subpopulations [12]-[15]. Li *et al.* [11] utilize a stochastic metapopulation model to simulate the spatiotemporal dynamics among 375 Chinese cities. The spatial spread of COVID-19 across cities is captured by the daily number of travelers from a city to another during the Spring Festival before the lockdown. Chinazzi *et al.* [16] model both the domestic (within Wuhan) and the international spread of the Coronavirus epidemic. The effects of the travel bans imposed in the city of Wuhan and the international travel ban adopted by several countries in early February 2020 are estimated. To model the international spread of the COVID-19 outbreak, the authors employ the stochastic global epidemic and mobility model. This metapopulation model is integrated with real-world data and relies on a network wherein each node represents a subpopulation located near major transportation hubs, e.g., airports; there are more than 3200 subpopulations, in roughly 200 different countries and territories. The degree of connection among subpopulations is represented by the number of people traveling daily among them. Within each subpopulation, there exist four states of the compartmental model, i.e., susceptible, latent (similar to exposed), infectious, and removed. The model generates an ensemble of possible epidemic scenarios described by the number of newly generated infections, time of disease onset in each subpopulation, and the number of traveling infection carriers.

### B. CONTRIBUTIONS AND PAPER ORGANIZATION

Most of the aforementioned epidemic models assume that relevant model parameters, e.g., the infection rate *β* and the recovery rate *γ*, are time-invariant, and several approaches have been proposed in the literature for tuning or estimating them [17]-[19]. However, the sudden imposition of restriction measures — and their subsequent relaxations — means that a *static* stochastic model is inappropriate. Moreover, even in the absence of dramatic restriction measures, there is no doubt that a time-varying model for the key epidemic parameters would better reflect the ground-truth.^2^

The main contribution of this article is to propose a Bayesian sequential learning and forecasting framework of the epidemic curve based on the data that authorities provide on a daily basis, e.g., number infected and number recovered. We leverage our recent research on unknown covariance matrix estimation [20] and self-tuning multisensor multitarget tracking [21]. Indeed, similarly to the target tracking problem, where the objective is to automatically detect the time instants when a target sharply maneuvers in order to improve the overall tracking performance, here we aim to closely track the epidemic curve and the model parameters in order to provide reliable and accurate forecast of the contagion. Adapting ideas and tools from those works, our approach to Bayesian sequential learning and forecasting of epidemic evolution is as follows. First, the model parameters are assumed to take on values from rich but prespecified finite sets; their time-evolution is modeled by Markov chains. Second, in order to capture the effects of mitigation strategies (e.g., mobility restriction, lockdown, wearing masks, and social distancing), the marginal posterior distributions of both the variable states (number of infected and recovered people) and model parameters (infection rate *β* and recovery rate *γ*), are calculated at each time by means of recursive prediction and update formulae. Finally, we develop an efficient implementation of the proposed method based on mixture models and provide a concrete example of application using the stochastic SIR model. The proposed method is validated on real datasets acquired during the recent COVID-19 outbreak in the Lombardia region, Italy, and in the USA. As we shall see, even adopting the simple stochastic SIR model, we obtain superior forecast accuracy when compared to prediction algorithms that use time-invariant parameter models. The approach developed in this article is general enough to be applied to more-sophisticated stochastic epidemiological models [8]-[10], including more-complex metapopulation models [11], [16]; these extensions are left for future investigations.

The remainder of the paper is organized as follows. In Section II, we describe a general Bayesian adaptive framework that can be tailored to any discrete-time epidemiological model, and in Section III we propose an implementation thereof based on the use of mixture models. In Section IV, we develop the mixture-based Bayesian sequential approach in the context of the stochastic SIR model. Section V presents results using synthetic as well as real data. Finally, in Section VI we provide some conclusions and possible directions for future investigations.

### C. NOTATION

Vectors are denoted by boldface lower-case letters (e.g., **a**), matrices by boldface upper-case letters (e.g., **A**), and sets by calligraphic letters (e.g., A). The transpose is written as (·)^T^. We write diag(*a*_1_, … , *a*_*N*_) for an *N* × *N* diagonal matrix with diagonal entries *a*_1_, … , *a*_*N*_, **I**_*N*_ for the *N* × *N* identity matrix, **1**_*N*_ for the *N*-dimensional vector of all ones, and **0** for the zero vector. E[⋅] denotes statistical expectation, and P(⋅) refers to both the probability density function (pdf) of a continuous random variable or vector and the probability mass function (pmf) of a discrete random variable or vector; the difference will be clear from the context. N(μ,C) indicates a Gaussian distributed vector with mean ***μ*** and covariance matrix **C**, and U(a,b) represents a uniformly distributed variable between *a* and *b*. Finally, N(x;μ,C) refers to a multivariate Gaussian pdf of random vector **x** with mean ***μ*** and covariance matrix **C**.

## II. PROPOSED ALGORITHM

We present a sequential Bayesian framework that, at each time interval, jointly computes the posterior distribution of *S* unknown time-varying states and of *M* unknown time-varying parameters. These unknown quantities are inferred at times *t*_*k*_, with *k* ∈ {1, 2, …}, using noisy observations (e.g., information on the number of infected, discharged COVID-19 patients from the hospitals, dead). We assume that the time interval Δ_*t*_ ≜ *t*_*k*_ – *t*_*k*–1_ between consecutive observations is one day, unless otherwise stated. We denote $x_{k}≜[x_{1,k},\ldots ,x_{S,k}{]}^{T}\in X\subseteq R^{S}$ as the state vector comprising the *S* epidemic states *x*_*s*,*k*_ at time *t*_*k*_ (e.g., numbers of infected and recovered individuals), and $\theta_{k}≜[\theta_{1,k},\ldots ,\theta_{M,k}{]}^{T}\in Q\subseteq R^{M}$ as the parameter vector comprising the *M* model parameters *θ*_*m*,*k*_ at time *t*_*k*_ (e.g., infection and recovery rates).

The objective of the proposed algorithm is twofold: to estimate, at each time *k*, the epidemic state vector **x**_*k*_ and the model parameter vector ***θ***_*k*_; and, at a fixed time *k*, to forecast the epidemic evolution up to time *k* + *K* with associated uncertainty in the form of prediction variance. Both tasks are based on the past and present observations. Hereafter, Section II-A describes the dynamic and observation models, and Section II-B and Section II-C present the proposed Bayesian sequential estimation and forecasting tasks, respectively. The reader who is already familiar with dynamic estimation of a *hybrid* state, that is, comprising the state of the dynamic model and nuisance parameters [20]-[22], might skip ahead to Section II-C.

### A. DYNAMIC AND OBSERVATION MODELS

The dynamic model that describes the evolution of the epidemic is formally expressed as

(1) $$x_{k}=f(\theta_{k},\theta_{k-1},x_{k-1};u_{k}),$$

where **u**_*k*_ is a random vector — whose dimension depends on *S*, *M*, and **f**(·) — with known distribution modeling the stochastic variation of the epidemic state in the time interval Δ_*t*_. Note that function **f**(·) might embed additional known (either time-varying or time-invariant) parameters. We assume that, conditioned on ***θ***_*k*_, ***θ***_*k*–1_, and **x**_*k*–1_, the state vector **x**_*k*_ is independent of the previous states and parameters, that is,

(2) $$P(x_{k}|\theta_{k},\theta_{k-1},x_{k-1},\theta_{k-2},x_{k-2},\ldots ,\theta_{1},x_{1})\;\;=P(x_{k}|\theta_{k},\theta_{k-1},x_{k-1}).$$

Given appropriate initial conditions, the pdf in (2) is fully determined by the dynamic model in (1) and the statistics of **u**_*k*_. Moreover, we assume that the parameter vector evolves according to a first-order Markov model fully described by the transition pdf $P(\theta_{k}|\theta_{k-1})$, assumed known. From these assumptions, it follows that the adopted Bayesian framework is a hierarchical Markov model (an event-driven dynamic process): firstly, the parameter vector evolves according to the Markov model described by $P(\theta_{k}|\theta_{k-1})$; then the state vector evolves, given the current parameter vector as well as the previous state and parameter vectors, according to (1). Furthermore, it is easy to verify that

$$P(x_{k},\theta_{k}|x_{k-1},\theta_{k-1},x_{k-2},\theta_{k-2},\ldots ,x_{1},\theta_{1})\;\;=P(x_{k},\theta_{k}|x_{k-1},\theta_{k-1}),$$

that is, the joint evolution of **x**_*k*_ and ***θ***_*k*_ follows a first-order Markov model.

The observation vector at time *k* is denoted by $z_{k}\in R^{B}$, and consists of up-to-date information on the state of the epidemic at time *k*. To take into account the randomness unavoidably present in real-word measurements, it is assumed that this information is *uncertain*, i.e., affected by noise (e.g., due to data collection errors, biases, holidays), and is modeled as

(3) $$z_{k}=h(x_{k},\theta_{k};v_{k}),$$

where **v**_*k*_ is a random vector with known distribution and whose dimension depends on *S*, *M*, *B*, and **h**(·). The model in (3) and the statistics of **v**_*k*_ determine the likelihood $P(z_{k}|x_{k},\theta_{k})$. For convenience, we define the vector $z_{1:k}≜[z_{1}^{T},\ldots ,z_{k}^{T}]$ containing all the observations up to time *k*, that is, past and present observations.

### B. BAYESIAN SEQUENTIAL ESTIMATION

The basic principles of Bayesian sequential estimation are now recalled. The reader is referred to [23] for further details. In the Bayesian setting, the estimation of state and parameters amounts to calculating the posterior pdf $P(x_{k}|z_{1:k})$ of the state vector **x**_*k*_, and the posterior pdf $P(\theta_{k}|z_{1:k})$ of the parameter vector ***θ***_*k*_, respectively. The minimum mean square estimators (MMSEs) of **x**_*k*_ and ***θ***_*k*_ are given by [23, Ch. 4]

(4) $${\hat{x}}_{k}^{E}≜\int x_{k}\;P(x_{k}|z_{1:k})dx_{k},$$

and

(5) $${\hat{\theta }}_{k}^{E}≜\int \theta_{k}\;P(\theta_{k}|z_{1:k})d\theta_{k},$$

respectively.^3^ We further note that, using the law of total probability, the pdf $P(x_{k}|z_{1:k})$ can be expressed as

(6) $$P(x_{k}|z_{1:k})=\int P(x_{k},\theta_{k}|z_{1:k})d\theta_{k}\;\;=\int P(x_{k}|\theta_{k},z_{1:k})P(\theta_{k}|z_{1:k})d\theta_{k}.$$

Thus, the estimation problem boils down to calculation of the posterior pdfs $P(x_{k}|\theta_{k},z_{1:k})$ and $P(\theta_{k}|z_{1:k})$. In the following, we show how they can be obtained sequentially through the implementation of recursive update and prediction steps.

#### 1) UPDATE STEP

Let us assume that we know the pdf of the parameter vector at time *k* given the observations up to time *k* – 1, i.e., $P(\theta_{k}|z_{1:k-1})$, and the pdf of the state vector at time *k* given the parameter vector at time *k* and the observations up to time *k* – 1, i.e., $P(x_{k}|\theta_{k},z_{1:k-1})$. Then, when a new observation **z**_*k*_ becomes available, the parameter vector pdf and the state vector pdf are updated through Bayes’ rule as

(7) $$P(\theta_{k}|z_{1:k})=\frac{P(z_{k},\theta_{k}|z_{1:k-1})}{\int P(z_{k},\theta_{k}^{\prime }|z_{1:k-1})d\theta_{k}^{\prime }}\;\;=\frac{P(z_{k}|\theta_{k},z_{1:k-1})P(\theta_{k}|z_{1:k-1})}{\int P(z_{k}|\theta_{k}^{\prime },z_{1:k-1})P(\theta_{k}^{\prime }|z_{1:k-1})d\theta_{k}^{\prime }},$$

and

(8) $$P(x_{k}|\theta_{k},z_{1:k})=\frac{P(z_{k},x_{k}|\theta_{k},z_{1:k-1})}{P(z_{k}|\theta_{k},z_{1:k-1})}\;\;=\frac{P(z_{k}|x_{k},\theta_{k},z_{1:k-1})P(x_{k}|\theta_{k},z_{1:k-1})}{P(z_{k}|\theta_{k},z_{1:k-1})}\;\;=\frac{P(z_{k}|x_{k},\theta_{k})P(x_{k}|\theta_{k},z_{1:k-1})}{P(z_{k}|\theta_{k},z_{1:k-1})},$$

respectively, where the last equality of (8) exploits the assumption that the observation at time *k* is conditionally independent of all the previous observations, given the state and parameter vectors at time *k*, i.e., $P(z_{k}|x_{k},\theta_{k},z_{1:k-1})=P(z_{k}|x_{k},\theta_{k})$. Using the same assumption and the law of total probability, the pdf $P(z_{k}|\theta_{k},z_{1:k-1})$ appearing in (7) is calculated as

(9) $$P(z_{k}|\theta_{k},z_{1:k-1})\;\;=\int P(z_{k},x_{k}|\theta_{k},z_{1:k-1})dx_{k}\;\;=\int P(z_{k}|x_{k},\theta_{k},z_{1:k-1})P(x_{k}|\theta_{k},z_{1:k-1})dx_{k}\;\;=\int P(z_{k}|x_{k},\theta_{k})P(x_{k}|\theta_{k},z_{1:k-1})dx_{k}.$$

#### 2) PREDICTION STEP

In the prediction step, we assume that the posterior pdf $P(\theta_{k}|z_{1:k})$ in (7) and the posterior pdf $P(x_{k}|\theta_{k},z_{1:k})$ in (8) are known, and derive the pdfs $P(\theta_{k+1}|z_{1:k})$ and $P(x_{k+1}|\theta_{k+1},z_{1:k})$. The former, i.e., the pdf of the predicted parameter vector at time *k* + 1, is obtained using the law of total probability and the Markovian assumption as follows:

(10) $$P(\theta_{k+1}|z_{1:k})=\int P(\theta_{k+1},\theta_{k}|z_{1:k})d\theta_{k}\;\;=\int P(\theta_{k+1}|\theta_{k})P(\theta_{k}|z_{1:k})d\theta_{k}.$$

Analogously, using the law of total probability, the pdf of the predicted state vector at time *k* + 1 is given by

(11) $$P(x_{k+1}|\theta_{k+1},z_{1:k})\;\;=\int P(x_{k+1},\theta_{k}|\theta_{k+1},z_{1:k})d\theta_{k}\;\;=\int P(x_{k+1}|\theta_{k+1},\theta_{k},z_{1:k})P(\theta_{k}|\theta_{k+1},z_{1:k})d\theta_{k}.$$

The first term within the integral (11), i.e., $P(x_{k+1}|\theta_{k+1},\theta_{k},z_{1:k})$, is calculated using the law of total probability and assuming that **x**_*k*+1_ is conditionally independent of **z**_1:*k*_ given ***θ***_*k*+1_, ***θ***_*k*_, and **x**_*k*_, i.e., $P(x_{k+1}|\theta_{k+1},\theta_{k},x_{k},z_{1:k})=P(x_{k+1}|\theta_{k+1},\theta_{k},x_{k})$. Thus,

(12) $$P(x_{k+1}|\theta_{k+1},\theta_{k},z_{1:k})\;\;=\int P(x_{k+1},x_{k}|\theta_{k+1},\theta_{k},z_{1:k})dx_{k}\;\;=\int P(x_{k+1}|\theta_{k+1},\theta_{k},x_{k})P(x_{k}|\theta_{k+1},\theta_{k},z_{1:k})dx_{k}\;\;=\int P(x_{k+1}|\theta_{k+1},\theta_{k},x_{k})P(x_{k}|\theta_{k},z_{1:k})dx_{k}.$$

The last step follows from the Markov property of the parameter vector. Indeed, the pdf $P(x_{k}|\theta_{k+1},\theta_{k},z_{1:k})$ can be calculated as

$$P(x_{k}|\theta_{k+1},\theta_{k},z_{1:k})\;\;=\frac{P(x_{k},\theta_{k+1},\theta_{k}|z_{1:k})}{P(\theta_{k+1},\theta_{k}|z_{1:k})}\;\;=\frac{P(\theta_{k+1}|x_{k},\theta_{k},z_{1:k})P(x_{k},\theta_{k}|z_{1:k})}{P(\theta_{k+1}|\theta_{k},z_{1:k})P(\theta_{k}|z_{1:k})}\;\;=\frac{P(\theta_{k+1}|x_{k},\theta_{k},z_{1:k})P(x_{k}|\theta_{k},z_{1:k})P(\theta_{k}|z_{1:k})}{P(\theta_{k+1}|\theta_{k},z_{1:k})P(\theta_{k}|z_{1:k})}\;\;=\frac{P(\theta_{k+1}|\theta_{k})P(x_{k}|\theta_{k},z_{1:k})P(\theta_{k}|z_{1:k})}{P(\theta_{k+1}|\theta_{k})P(\theta_{k}|z_{1:k})}\;\;=P(x_{k}|\theta_{k},z_{1:k}).$$

The second term within the integral (11), i.e., $P(\theta_{k}|\theta_{k+1},z_{1:k})$, is obtained using Bayes’ rule and exploiting again the Markov property of the parameter vector, that is,

(13) $$P(\theta_{k}|\theta_{k+1},z_{1:k})=\frac{P(\theta_{k+1},\theta_{k}|z_{1:k})}{P(\theta_{k+1}|z_{1:k})}\;\;=\frac{P(\theta_{k+1}|\theta_{k},z_{1:k})P(\theta_{k}|z_{1:k})}{P(\theta_{k+1}|z_{1:k})}\;\;=\frac{P(\theta_{k+1}|\theta_{k})P(\theta_{k}|z_{1:k})}{P(\theta_{k+1}|z_{1:k})},$$

where $P(\theta_{k+1}|z_{1:k})$ is given by (10).

### C. FORECASTING

As the model we consider is nonlinear, the forecast of the epidemic evolution is assessed numerically through a methodology known as ensemble forecasting [24]-[28], that consists of generating a collection of possible evolutions of the epidemic — given the state and parameter vectors estimated so far — and provide a single mean forecast with associated uncertainty. Let us assume that the latest available observation is **z**_*k*_, and that the posterior pdfs $P(\theta_{k}|z_{1:k})$ and $P(x_{k}|z_{1:k})=\int P(x_{k}|\theta_{k},z_{1:k})P(\theta_{k}|z_{1:k})d\theta_{k}$ (cf. (6)) are known; the proposed ensemble forecasting approach is a Monte-Carlo technique that samples the posterior distribution of **x**_*k*_ and ***θ***_*k*_, and evolves these sampled initial vectors up to time *k* + *K*, where *K* is the forecast horizon. Specifically, let ${\tilde{x}}_{k}^{\left(j\right)}$ and ${\tilde{\theta }}_{k}^{\left(j\right)}$ be the *j*^th^ state vector and parameter vector samples extracted from $P(x_{k}|z_{1:k})$ and $P(\theta_{k}|z_{1:k})$, respectively, where j∈J≜{1,…,J} and *J* is the ensemble size. The sampled state and parameter vectors are then allowed to evolve^4^ according to the state vector forecast transition distribution $P_{F}\left({\tilde{x}}_{k^{\prime }}^{\left(j\right)}|{\tilde{\theta }}_{k^{\prime }}^{\left(j\right)},{\tilde{\theta }}_{k^{\prime }-1}^{\left(j\right)},{\tilde{x}}_{k^{\prime }-1}^{\left(j\right)}\right)$ and the parameter vector forecast transition distribution $P_{F}\left({\tilde{\theta }}_{k^{\prime }}^{\left(j\right)}|{\tilde{\theta }}_{k^{\prime }-1}^{\left(j\right)}\right)$, respectively, for *k*′ ∈ {*k* + 1, … , *k* + *K*}. We observe that these transition distributions can be equal to the transition distributions used within the Bayesian sequential estimation procedure described in the previous section, that is, $P(x_{k}|\theta_{k},\theta_{k-1},x_{k-1})$ and $P(\theta_{k}|\theta_{k-1})$, respectively, or can be suitably devised to improve the forecast performance. Finally, defining the ensemble state matrix as ${\tilde{X}}_{k}≜[{\tilde{x}}_{k}^{\left(1\right)},\ldots ,{\tilde{x}}_{k}^{\left(J\right)}]\in X^{1\times J}\;\subseteq \;R^{S\times J}$, and the ensemble parameter matrix as ${\tilde{\Theta }}_{k}≜[{\tilde{\theta }}_{k}^{\left(1\right)},\ldots ,{\tilde{\theta }}_{k}^{\left(J\right)}]\in Q^{1\times J}\;\subseteq \;R^{M\times J}$, the mean of the epidemic state and model parameter at any time step *k*′ ∈ {*k* + 1, … , *k* + *K*} can be calculated as sample means of ${\tilde{X}}_{k^{\prime }}$ and ${\tilde{\Theta }}_{k^{\prime }}$, respectively; that is [29],

(14) $${\hat{x}}_{k^{\prime }}^{F}≜\frac{1}{J}{\tilde{X}}_{k^{\prime }}\cdot 1_{J},\;\;{\hat{\theta }}_{k^{\prime }}^{F}≜\frac{1}{J}{\tilde{\Theta }}_{k^{\prime }}\cdot 1_{J}.$$

Higher order moments, such as sample covariance matrices, can also be computed [29].

## III. MIXTURE MODEL IMPLEMENTATION

This section describes a mixture model implementation — similar to the approach proposed in [20] — of the Bayesian sequential estimation procedure presented in Section II-B. For computational efficiency, the first step is the discretization of the parameter vector ***θ***_*k*_ = [*θ*_1,*k*_, … , *θ*_*M*,*k*_]^T^, such that each element *θ*_*m*,*k*_, *m* ∈ {1, … , *M*}, takes on values from a finite set $D_{m}≜\{\vartheta_{1}^{\left(m\right)},\ldots ,\vartheta_{D_{m}}^{\left(m\right)}\}$. It follows that $\theta_{k}\in D$, where $D≜D_{1}\times \cdots \times D_{M}$ is the discretized finite set with cardinality $D≜\prod_{m=1}^{M}D_{m}$. We note that all the expressions in Section II-B remain valid, provided that integrals ∫ d***θ*** are replaced with summations Σ_***θ***_; e.g., the MMSE estimator of ***θ***_*k*_ in (5) is rewritten as

(15) $${\hat{\theta }}_{k}^{E}≜\sum_{\theta_{k}\in D}\theta_{k}\;P(\theta_{k}|z_{1:k}).$$

The key aspect of the formulation is to model the pdf $P(x_{k}|\theta_{k},z_{1:k-1})$ as a mixture of *N* components, that is,

(16) $$P(x_{k}|\theta_{k},z_{1:k-1})=\sum_{n=1}^{N}w_{k|k-1}^{\left(n,\theta_{k}\right)}\;P(x_{k}|N_{k}=n,\theta_{k},z_{1:k-1}),$$

where $w_{k|k-1}^{\left(n,\theta_{k}\right)}≜P(N_{k}=n|\theta_{k},z_{1:k-1})$ and $P(x_{k}|N_{k}=n,\theta_{k},z_{1:k-1})$ are weight and pdf of the *n*^th^ component, respectively. The auxiliary variable *N*_*k*_ ∈ {1, … , *N*} models the switch between the *N* mixture components; hereafter, for notational convenience, we will simply use *n* to denote that *N*_*k*_ takes on the value *n*, i.e., *N*_*k*_ = *n*. In the next two subsections, exploiting the development presented in Section II-B1 and Section II-B2, we provide the expressions for the sequential update and prediction of the state and parameter vectors according to the mixture model.

### A. UPDATE STEP

The posterior pdf $P(x_{k}|\theta_{k},z_{1:k})$ appearing in (8) can be written through the law of total probability as

(17) $$P(x_{k}|\theta_{k},z_{1:k})=\sum_{n=1}^{N}w_{k|k}^{\left(n,\theta_{k}\right)}\;P(x_{k}|n,\theta_{k},z_{1:k}).$$

The updated weight $w_{k|k}^{\left(n,\theta_{k}\right)}\;=\;P(n|\theta_{k},z_{1:k})$ is calculated through Bayes’s rule as

(18) $$w_{k|k}^{\left(n,\theta_{k}\right)}=\frac{\alpha_{k|k-1}^{\left(n,\theta_{k}\right)}w_{k|k-1}^{\left(n,\theta_{k}\right)}}{\sum_{n^{\prime }=1}^{N}\alpha_{k|k-1}^{\left(n^{\prime },\theta_{k}\right)}w_{k|k-1}^{\left(n^{\prime },\theta_{k}\right)}},$$

where the update coefficient $\alpha_{k|k-1}^{\left(n,\theta_{k}\right)}\;≜\;P(z_{k}|n,\theta_{k},z_{1:k-1})$ can be derived from (9) assuming the conditional independence of the observation **z**_*k*_ from the previous observations **z**_1:*k*–1_ and the specific mixand *n*, given **x**_*k*_ and ***θ***_*k*_, i.e., $P(z_{k}|n,x_{k},\theta_{k},z_{1:k-1})=P(z_{k}|x_{k},\theta_{k})$. That is,

(19) $$\alpha_{k|k-1}^{\left(n,\theta_{k}\right)}=P(z_{k}|n,\theta_{k},z_{1:k-1})\;\;=\int P(z_{k}|x_{k},\theta_{k})P(x_{k}|n,\theta_{k},z_{1:k-1})dx_{k}.$$

Using the same assumption, the posterior pdf of the *n*^th^ mixture component is calculated from (8) as

(20) $$P(x_{k}|n,\theta_{k},z_{1:k})=\frac{P(z_{k}|x_{k},\theta_{k})P(x_{k}|n,\theta_{k},z_{1:k-1})}{\alpha_{k|k-1}^{\left(n,\theta_{k}\right)}}.$$

Then, using (7) with integrals replaced by summations, the posterior pmf of the parameter vector is obtained as

(21) $$P(\theta_{k}|z_{1:k})=\frac{P(\theta_{k}|z_{1:k-1})P(z_{k}|\theta_{k},z_{1:k-1})}{\sum_{\theta_{k}^{\prime }\in D}P(\theta_{k}^{\prime }|z_{1:k-1})P(z_{k}|\theta_{k}^{\prime },z_{1:k-1})},$$

where, through the law of total probability, $P(z_{k}|\theta_{k},z_{1:k-1})$ can be calculated as

$$P(z_{k}|\theta_{k},z_{1:k-1})\;\;=\sum_{n=1}^{N}P(z_{k},n|\theta_{k},z_{1:k-1})\;\;=\sum_{n=1}^{N}P(z_{k}|n,\theta_{k},z_{1:k-1})P(n|\theta_{k},z_{1:k-1}).$$

Here, we recognize the update coefficient $\alpha_{k|k-1}^{\left(n,\theta_{k}\right)}\;=\;P(z_{k}|n,\theta_{k},z_{1:k-1})$ and the weight $w_{k|k-1}^{\left(n,\theta_{k}\right)}=P(n|\theta_{k},z_{1:k-1})$. Therefore, the posterior pmf in (21) can be finally recast as

(22) $$P(\theta_{k}|z_{1:k})=\frac{P(\theta_{k}|z_{1:k-1})\sum_{n=1}^{N}\alpha_{k|k-1}^{\left(n,\theta_{k}\right)}w_{k|k-1}^{\left(n,\theta_{k}\right)}}{\sum_{\theta_{k}^{\prime }\in D}P(\theta_{k}^{\prime }|z_{1:k-1})\sum_{n^{\prime }=1}^{N}\alpha_{k|k-1}^{\left(n^{\prime },\theta_{k}^{\prime }\right)}w_{k|k-1}^{\left(n^{\prime },\theta_{k}^{\prime }\right)}}.$$

### B. PREDICTION STEP

The pmf of the predicted parameter vector at time *k* + 1, i.e., $P(\theta_{k+1}|z_{1:k})$, is simply obtained by inserting (22) into (10), where the integral is replaced by summation. To derive the pdf of the predicted state vector at time *k* + 1, i.e., $P(x_{k+1}|\theta_{k+1},z_{1:k})$, instead, we consider the discrete version of (11), that is,

$$P(x_{k+1}|\theta_{k+1},z_{1:k})\;\;=\sum_{\theta_{k}\in D}P(\theta_{k}|\theta_{k+1},z_{1:k})P(x_{k+1}|\theta_{k+1},\theta_{k},z_{1:k}),$$

and insert therein (12) and (13) to obtain

(23) $$P(x_{k+1}|\theta_{k+1},z_{1:k})\;\;=\sum_{\theta_{k}\in D}\frac{P(\theta_{k+1}|\theta_{k})P(\theta_{k}|z_{1:k})}{P(\theta_{k+1}|z_{1:k})}\;\;\times \int P(x_{k+1}|\theta_{k+1},\theta_{k},x_{k})P(x_{k}|\theta_{k},z_{1:k})dx_{k}.$$

Finally, by using (17) into (23), the latter can be recast as

$$P(x_{k+1}|\theta_{k+1},z_{1:k})\;\;=\sum_{n=1}^{N}\sum_{\theta_{k}\in D}w_{k+1|k}^{\left(n,\theta_{k+1},\theta_{k}\right)}P(x_{k+1}|n,\theta_{k+1},\theta_{k},z_{1:k}),$$

where the pdf of the *n*^th^ component, i.e., $P(x_{k+1}|n,\theta_{k+1},\theta_{k},z_{1:k})$, is

(24) $$P(x_{k+1}|n,\theta_{k+1},\theta_{k},z_{1:k})\;\;≜\int P(x_{k+1}|\theta_{k+1},\theta_{k},x_{k})P(x_{k}|n,\theta_{k},z_{1:k})dx_{k},$$

and the predicted weights are defined as

(25) $$w_{k+1|k}^{\left(n,\theta_{k+1},\theta_{k}\right)}≜\frac{P(\theta_{k+1}|\theta_{k})P(\theta_{k}|z_{1:k})}{P(\theta_{k+1}|z_{1:k})}w_{k|k}^{\left(n,\theta_{k}\right)}.$$

We note that in the prediction step the number of mixture components increases from *N* to *N* × *D*, thus a suitable merging/pruning criterion is required to avoid the exponential growth of the computational complexity [30], [31].

*Remark:* We aim to provide a general framework for adaptive Bayesian estimation of epidemic evolution, here implemented via an efficient mixture model. Nonetheless, the same nonlinear problem could have been approached and implemented differently, for example by using an extended or unscented Kalman filter (EKF or UKF), or by means of sequential Monte Carlo (SMC) methods, e.g., particle filters [32]. These are not alternate to our approach, but can be possibly combined, as noted later in Section IV-B. However, we also observe that direct use of the aforementioned techniques — if not adequately tailored to the specific problem — may lead to poor or unexpected results. For example, the EKF approximation of the nonlinear behaviour of the system through local linearization, might fail in the presence of strong nonlinearities, leading to unreliable estimates or even to divergence. The UKF can potentially provide higher-order estimation accuracy using the unscented transform, but this usually has the effect of simply delaying the unavoidable divergence that will still happen in the case of severe process or measurement nonlinearities. On the other hand, SMC methods generally provide reliable numerical approximations to sequential nonlinear estimation problems. However, in real-world applications, where the system also depends on unknown time-varying parameters to be inferred from uncertain data, conventional particle filters could fail to detect and track the change in parameters, quickly leading to implementation issues, such as sample impoverishment. In addition, high performance of particle methods comes at the expense of increased computational demands.

Another class of methods is based on system identification/machine learning (ML) techniques. Canonical approaches here include expectation-maximization, variational Bayes methods, and the variety of nonlinear auto-regressive models with external inputs, recurrent neural networks, long short-term memory networks [33]-[36]. The non-parametric ML methods suffer from lack of explainability and causal reasoning needed for policy decisions in pandemics.

## IV. ESTIMATION AND FORECASTING WITH STOCHASTIC SIR MODEL

### A. DYNAMIC AND OBSERVATION MODEL

The SIR model [4], [5] subdivides the population of a community into three interacting groups: susceptible, infectious, and recovered individuals. The interactions are governed by the infection rate, usually denoted by *β*, that is the average rate at which an infected individual can infect a susceptible one, and by the recovery rate, generally called *γ*. Let *P* be the total population size^5^; *s*_*k*_, *i*_*k*_, and *r*_*k*_ be the normalized (to *P*) number of susceptible, infectious, and recovered individuals at time *k*, such that *s*_*k*_ + *i*_*k*_ + *r*_*k*_ = 1; and *β*_*k*_ and *γ*_*k*_ be the infection and recovery rates at time *k*. The discrete-time stochastic SIR system of equations is expressed as [17], [37]

$$s_{k}=s_{k-1}-\beta_{k-1}s_{k-1}i_{k-1}\Delta_{t}+\sigma_{1,k-1}u_{1,k},\;i_{k}=i_{k-1}+\beta_{k-1}s_{k-1}i_{k-1}\Delta_{t}-\gamma_{k-1}i_{k-1}\Delta_{t}\;\;-\sigma_{1,k-1}u_{1,k}+\sigma_{2,k-1}u_{2,k},\;r_{k}=r_{k-1}+\gamma_{k-1}i_{k-1}\Delta_{t}-\sigma_{2,k-1}u_{2,k},$$

where $\sigma_{1,k}≜\sqrt{P^{-1}\beta_{k}s_{k}i_{k}}$, $\sigma_{2,k}≜\sqrt{P^{-1}\gamma_{k}i_{k}}$, and $u_{k}≜[u_{1,k},u_{2,k}{]}^{T}∼N(0,I_{2}\Delta_{t})$. Since *s*_*k*_ and *i*_*k*_ determine *r*_*k*_, we define the state vector as **x**_*k*_ ≜ [*s*_*k*_, *i*_*k*_]^T^, and the parameter vector as ***θ***_*k*_ ≜ [*β*_*k*_, *γ*_*k*_]^T^; hence, we have *S* = 2 and *M* = 2. The dynamic model of the epidemic is then expressed as (cf. (1))

(26) $$x_{k}=f_{1}(x_{k-1},\theta_{k-1})+f_{2}(x_{k-1},\theta_{k-1})u_{k}$$

where

$$f_{1}(x_{k},\theta_{k})≜\left[\begin{array}{c} (1-\beta_{k}i_{k}\Delta_{t})s_{k}\\ (1+\beta_{k}s_{k}\Delta_{t}-\gamma_{k}\Delta_{t})i_{k} \end{array}\right],$$

and

$$f_{2}(x_{k},\theta_{k})≜\left[\begin{array}{cc} \sqrt{P^{-1}\beta_{k}s_{k}i_{k}} & 0\\ -\sqrt{P^{-1}\beta_{k}s_{k}i_{k}} & \sqrt{P^{-1}\gamma_{k}i_{k}} \end{array}\right].$$

From (26) it then follows that the state transition pdf (cf. (2)) is independent of the current parameter vector ***θ***_*k*_, i.e., $P(x_{k}|\theta_{k},\theta_{k-1},x_{k-1})=P(x_{k}|\theta_{k-1},x_{k-1})$, and is distributed according to

(27) $$P(x_{k}|\theta_{k-1},x_{k-1})\;\;=N(x_{k};f_{1}(x_{k-1},\theta_{k-1}),F(x_{k-1},\theta_{k-1})\Delta_{t}),$$

where

$$F(x_{k},\theta_{k})=\left[\begin{array}{cc} \sigma_{1,k}^{2} & -\sigma_{1,k}^{2}\\ -\sigma_{1,k}^{2} & \sigma_{1,k}^{2}+\sigma_{2,k}^{2} \end{array}\right].$$

We observe the (uncertain) normalized number of infected and of recovered individuals at each time *k*. Therefore, *B* = 2, and the observation model (cf. (3)) is

$$z_{k}=h_{1}(x_{k})+v_{k},$$

where $v_{k}∼N(0,R(x_{k}))$ models the observation uncertainty, and

$$h_{1}(x_{k})≜[0\;\;1{]}^{T}+Hx_{k},\;\;H≜\left[\begin{array}{cc} 0 & 1\\ -1 & -1 \end{array}\right].$$

The covariance matrix **R**(**x**_*k*_) depends on the state vector at time *k*: since we assume that the observation “noise” accrues from the sum of uncertainties of each individual epidemic state, the variances are linear in the number of infected and recovered individuals, respectively. Hence, we define

(28) $$R(x_{k})≜\left[\begin{array}{cc} P^{-1}i_{k} & 0\\ 0 & P^{-1}(1-i_{k}-s_{k}) \end{array}\right]R_{c},$$

where **R**_c_ is a constant diagonal matrix. Thus, the likelihood is independent of ***θ***_*k*_, i.e., $P(z_{k}|x_{k},\theta_{k})=P(z_{k}|x_{k})$, and distributed according to

(29) $$P(z_{k}|x_{k})=N(z_{k};h_{1}(x_{k}),R(x_{k})).$$

### B. GAUSSIAN MIXTURE FILTER

Given the Gaussian nature of the dynamic and observation models, we adopt a Gaussian mixture implementation of the Bayesian sequential estimation. That is, we assume that the pdf of the *n*^th^ mixture component in (16) is Gaussian with mean ${\hat{x}}_{k|k-1}^{\left(n,\theta_{k}\right)}$ and covariance matrix ${\hat{C}}_{k|k-1}^{\left(n,\theta_{k}\right)}$, i.e.,

(30) $$P(x_{k}|n,\theta_{k},z_{1:k-1})=N\left(x_{k};{\hat{x}}_{k|k-1}^{\left(n,\theta_{k}\right)},{\hat{C}}_{k|k-1}^{\left(n,\theta_{k}\right)}\right).$$

When the observation **z**_*k*_ is available, mean, covariance matrix, and weight are updated. Specifically, the weight of the *n*^th^ mixand is updated as in (18) through the coefficient $\alpha_{k|k-1}^{\left(n,\theta_{k}\right)}$; this, in turn, is calculated recalling that the likelihood in (29) is independent of ***θ***_k_, and inserting (29) and (30) into (19), that is,

(31) $$\alpha_{k|k-1}^{\left(n,\theta_{k}\right)}\;=\int N\left(z_{k};h_{1}(x_{k}),R(x_{k})\right)N\left(x_{k};{\hat{x}}_{k|k-1}^{\left(n,\theta_{k}\right)},{\hat{C}}_{k|k-1}^{\left(n,\theta_{k}\right)}\right)dx_{k}\;\approx N\left(z_{k};h_{1}\left({\hat{x}}_{k|k-1}^{\left(n,\theta_{k}\right)}\right),R\left({\hat{x}}_{k|k-1}^{\left(n,\theta_{k}\right)}\right)+H{\hat{C}}_{k|k-1}^{\left(n,\theta_{k}\right)}H^{T}\right),$$

where we made the approximation $R(x_{k})\approx R({\hat{x}}_{k|k-1}^{\left(n,\theta_{k}\right)})$. We observe that the last step would be an equality — rather than an approximation — if the observation covariance matrix was independent of the state **x**_*k*_. Then, the updated pdf of the *n*^th^ Gaussian component is obtained by inserting (29), (30), and (31) into (20), and is equal to [30]

(32) $$P(x_{k}|n,\theta_{k},z_{1:k})=N\left(x_{k};{\hat{x}}_{k|k}^{\left(n,\theta_{k}\right)},{\hat{C}}_{k|k}^{\left(n,\theta_{k}\right)}\right),$$

where

$${\hat{x}}_{k|k}^{\left(n,\theta_{k}\right)}={\hat{x}}_{k|k-1}^{\left(n,\theta_{k}\right)}+K_{k|k-1}^{\left(n,\theta_{k}\right)}\left[z_{k}-h_{1}\left({\hat{x}}_{k|k-1}^{\left(n,\theta_{k}\right)}\right)\right],\;{\hat{C}}_{k|k}^{\left(n,\theta_{k}\right)}={\hat{C}}_{k|k-1}^{\left(n,\theta_{k}\right)}-K_{k|k-1}^{\left(n,\theta_{k}\right)}\;H\;{\hat{C}}_{k|k-1}^{\left(n,\theta_{k}\right)},$$

and

$$K_{k|k-1}^{\left(n,\theta_{k}\right)}={\hat{C}}_{k|k-1}^{\left(n,\theta_{k}\right)}\;H^{T}{\left[H\;{\hat{C}}_{k|k-1}^{\left(n,\theta_{k}\right)}\;H^{T}+R\left({\hat{x}}_{k|k-1}^{\left(n,\theta_{k}\right)}\right)\right]}^{-1}.$$

This is similar to a standard Kalman update, per mixture element, with essential difference that the measurement noise covariance is state-dependent.

Let us now consider the evolution of the mixands according to the dynamic model. The *n*^th^ predicted weight is computed using (25) where, assuming that infection rate *β*_*k*_ and recovery rate *γ*_*k*_ evolve independently, the parameter vector transition pmf can be written as $P(\theta_{k+1}|\theta_{k})=P(\beta_{k+1}|\beta_{k})P(\gamma_{k+1}|\gamma_{k})$; the marginal transition pmfs $P(\beta_{k+1}|\beta_{k})$ and $P(\gamma_{k+1}|\gamma_{k})$ are specified later in this section. The pdf of the *n*^th^ predicted mixand is obtained by recalling that the transition pdf in (27) is independent of the current parameter vector ***θ***_*k*_, and inserting (27) and (32) into (24). This yields

(33) $$P(x_{k+1}|n,\theta_{k+1},\theta_{k},z_{1:k})\;\;=\int N(x_{k+1};f_{1}(x_{k},\theta_{k}),F(x_{k},\theta_{k})\Delta_{t})\;\;\times N\left(x_{k};{\hat{x}}_{k|k}^{\left(n,\theta_{k}\right)},{\hat{C}}_{k|k}^{\left(n,\theta_{k}\right)}\right)dx_{k}.$$

Given the nonlinearity of the dynamic model (26), the integral (33) cannot be computed explicitly. A viable alternative is to approximate the pdf $P(x_{k+1}|n,\theta_{k+1},\theta_{k},z_{1:k})=P(x_{k+1}|n,\theta_{k},z_{1:k})$ as a Gaussian via moment matching, that is,

(34) $$P(x_{k+1}|n,\theta_{k},z_{1:k})=N\left(x_{k+1};{\hat{x}}_{k+1|k}^{\left(n,\theta_{k}\right)},{\hat{C}}_{k+1|k}^{\left(n,\theta_{k}\right)}\right).$$

The computation of ${\hat{x}}_{k+1|k}^{\left(n,\theta_{k}\right)}$ and of ${\hat{C}}_{k+1|k}^{\left(n,\theta_{k}\right)}$ by moment matching is detailed in Appendix A. An alternative method to solve integral (33) is via the unscented transformation used within the UKF.

As described in Section III, the parameter vector ***θ***_*k*_ = [*β*_*k*_, *γ*_*k*_]^T^ is discretized for computational efficiency. Specifically, $\beta_{k}\;\in \;D_{1}=\{\vartheta_{1}^{\left(1\right)},\ldots ,\vartheta_{D_{1}}^{\left(1\right)}\}$ and $\gamma_{k}\;\in \;D_{2}=\{\vartheta_{1}^{\left(2\right)},\ldots ,\vartheta_{D_{2}}^{\left(2\right)}\}$. For concreteness, we assume that $D_{m}$ (*m* = 1 or 2) is an ordered set, i.e., such that for any *j*, ℓ ∈ {1, … , *D*_*m*_} with *j* < ℓ, we have $\vartheta_{j}^{\left(m\right)}<\vartheta_{\ell }^{\left(m\right)}$; and that the elements of $D_{m}$ are selected to be equally spaced between $\vartheta_{1}^{\left(m\right)}$ and $\vartheta_{D_{m}}^{\left(m\right)}$. The marginal transition pmfs $P(\beta_{k}|\beta_{k-1})$ and $P(\gamma_{k}|\gamma_{k-1})$ are therefore fully described by the matrix **P**_*β*_ ∈ [0, 1]^*D*_1_×*D*_1_^ and matrix **P**_*γ*_ ∈ [0, 1]^*D*_2_×*D*_2_^, respectively, where

(35) $$[P_{\beta }{]}_{j,\ell }≜P(\beta_{k}=\vartheta_{j}^{\left(1\right)}|\beta_{k-1}=\vartheta_{\ell }^{\left(1\right)}),$$

and

(36) $$[P_{\gamma }{]}_{j,\ell }≜P(\gamma_{k}=\vartheta_{j}^{\left(2\right)}|\gamma_{k-1}=\vartheta_{\ell }^{\left(2\right)}).$$

We note that $\sum_{j=1}^{D_{1}}[P_{\beta }{]}_{j,\ell }=1$ and that $\sum_{j=1}^{D_{2}}[P_{\gamma }{]}_{j,\ell }=1$. Eventually, according to (4) and (6), and replacing the integral in (6) with the summation, the MMSE estimates of the normalized numbers of susceptible and infectious are

(37) $${\hat{s}}_{k}^{E}=\sum_{\beta_{k}\in D_{1}}\sum_{\gamma_{k}\in D_{2}}P(\beta_{k},\gamma_{k}|z_{1:k})\;\;\times \iint s_{k}P(s_{k},i_{k}|\beta_{k},\gamma_{k},z_{1:k})di_{k}ds_{k},$$

and

(38) $${\hat{ı}}_{k}^{E}=\sum_{\beta_{k}\in D_{1}}\sum_{\gamma_{k}\in D_{2}}P(\beta_{k},\gamma_{k}|z_{1:k})\;\;\times \iint i_{k}P(s_{k},i_{k}|\beta_{k},\gamma_{k},z_{1:k})ds_{k}di_{k},$$

respectively; and, according to (15), the MMSE estimates of the infection and recovery rates are

(39) $${\hat{\beta }}_{k}^{E}=\sum_{\beta_{k}\in D_{1}}\beta_{k}\sum_{\gamma_{k}\in D_{2}}P(\beta_{k},\gamma_{k}|z_{1:k}),$$

and

(40) $${\hat{\gamma }}_{k}^{E}=\sum_{\gamma_{k}\in D_{2}}\gamma_{k}\sum_{\beta_{k}\in D_{1}}P(\beta_{k},\gamma_{k}|z_{1:k}),$$

respectively. Note that the estimates for the parameters ${\hat{\beta }}_{k}^{E}$ and ${\hat{\gamma }}_{k}^{E}$ are updated automatically based on the attractiveness (measured in terms of the relative likelihoods) of the estimates that assume them. Finally, the prior pmfs of the infection and recovery rates at time *k* = 0 are set to $P(\beta_{0})=N(\beta_{0};{\bar{\beta }}_{0},\sigma_{\beta }^{2})$ and $P(\gamma_{0})=N(\gamma_{0};{\bar{\gamma }}_{0},\sigma_{\gamma }^{2})$; the prior pdf of the *n*^th^ Gaussian component at time *k* = 0 is $P(x_{0}|n,\theta_{0})=P(x_{0}|n)=N(x_{0};{\bar{x}}_{0}^{\left(n\right)},{\bar{C}}_{0}^{\left(n\right)})$.

A detailed statement of the proposed Gaussian mixture filter for the Bayesian estimation of the epidemic evolution with the stochastic SIR model is provided in Algorithm 1.

### C. FORECASTING

The forecasting is as described in Section II-C. Let us assume that **z**_*k*_ is the most recent available observation, and that the posterior pdf $P(x_{k}|z_{1:k})$ and pmf $P(\theta_{k}|z_{1:k})$ are known.

The *j*^th^ sample state vector extracted from the posterior pdf $P(x_{k}|z_{1:k})$ is ${\tilde{x}}_{k}^{\left(j\right)}=[{\tilde{s}}_{k}^{\left(j\right)},{\tilde{ı}}_{k}^{\left(j\right)}{]}^{T}$, j∈J, and evolves according to the state vector forecast transition distribution $P_{F}\left({\tilde{x}}_{k}^{\left(j\right)}|{\tilde{\theta }}_{k}^{\left(j\right)},{\tilde{\theta }}_{k-1}^{\left(j\right)},{\tilde{x}}_{k-1}^{\left(j\right)}\right)$; we assume that this forecast transition distribution coincides with that used within the sequential Bayesian estimation procedure (cf. (27)), i.e., we assume that the sampled state vector ${\tilde{x}}_{k}^{\left(j\right)}$ evolves according to the dynamic model in (26).

Concerning the parameter vectors, in order to obtain samples ${\tilde{\theta }}_{k}^{\left(j\right)}=[{\tilde{\beta }}_{k}^{\left(j\right)},{\tilde{\gamma }}_{k}^{\left(j\right)}{]}^{T}$, j∈J, from the infinite set Q — rather than the discrete finite set D —, the posterior pmf $P(\theta_{k}|z_{1:k})$ is approximated with a suitable continuous distribution; here, $P(\theta_{k}|z_{1:k})$ is approximated with a bivariate Gaussian pdf and samples ${\tilde{\theta }}_{k}^{\left(j\right)}$ are extracted from it. Then, these sampled parameter vectors are allowed to evolve according to the parameter vector forecast transition distribution $P_{F}\left({\tilde{\theta }}_{k}^{\left(j\right)}|{\tilde{\theta }}_{k-1}^{\left(j\right)}\right)=P_{F}\left({\tilde{\beta }}_{k}^{\left(j\right)}|{\tilde{\beta }}_{k-1}^{\left(j\right)}\right)P_{F}\left({\tilde{\gamma }}_{k}^{\left(j\right)}|{\tilde{\gamma }}_{k-1}^{\left(j\right)}\right)$, where we assumed the infection and recovery rates to change independently. We recall that the inverse of the recovery rate expresses the average time that an individual takes to move from the group of infected (I) to the group of recovered (R) people; in our model, the latter includes both those discharged from hospitals, and those for whom the infection was fatal. Even though it is likely that the recovery rate will change during the forecast period (due to, e.g., reporting delays or the application of different criteria used to declare an individual recovered), there are no prior information that would suggest when and how this will happen; it is therefore reasonable to assume the recovery rate to be constant and deterministic during the forecast period. This equals to set the recovery rate forecast transition distribution to

$$P_{F}({\tilde{\gamma }}_{k}^{\left(j\right)}|{\tilde{\gamma }}_{k-1}^{\left(j\right)})=\delta ({\tilde{\gamma }}_{k}^{\left(j\right)}-{\tilde{\gamma }}_{k-1}^{\left(j\right)}),$$

where *δ*(·) is the Dirac delta. The infection rate, instead, models the interaction between people, and it is therefore affected by the restriction measures. Therefore, once its time evolution is captured, it is reasonable to assume — in the absence of further knowledge — that it keeps the same trend linearly. That is, the infection rate samples ${\tilde{\beta }}_{k}^{\left(j\right)}$, j∈J, are assumed to evolve according to

$${\tilde{\beta }}_{k^{\prime }}^{\left(j\right)}={\tilde{\beta }}_{k^{\prime }-1}^{\left(j\right)}+{\dot{\beta }}_{k}\Delta_{t}+d_{L,k}\;,$$

for *k*′ ∈ {*k* + 1, … , *k* + *K*}, where ${\dot{\beta }}_{k}$ is a constant slope, and $d_{L,k}\;∼\;N(0,{\dot{\sigma }}_{L,k}^{2})$; hence, the infection rate forecast transition distribution is

$$P_{F}({\tilde{\beta }}_{k}^{\left(j\right)}|{\tilde{\beta }}_{k-1}^{\left(j\right)})=N({\tilde{\beta }}_{k}^{\left(j\right)};{\tilde{\beta }}_{k-1}^{\left(j\right)}+{\dot{\beta }}_{k}\Delta_{t},{\dot{\sigma }}_{L,k}^{2}).$$

Appendix B provides details on the estimation of the slope ${\dot{\beta }}_{k}$ over the time interval [*k* — *L*, *k*], on the selection of *L*, with *L*_min_ ⩽ *L* ⩽ *L*_max_, and on the computation of ${\dot{\sigma }}_{L,k}^{2}$.

Eventually, defining the ensemble state and parameter matrices as ${\tilde{X}}_{k}=[{\tilde{s}}_{k},{\tilde{ı}}_{k}]$ and ${\tilde{\Theta }}_{k}=[{\tilde{\beta }}_{k},{\tilde{\gamma }}_{k}]$, respectively, where ${\tilde{s}}_{k}≜[{\tilde{s}}_{k}^{\left(1\right)},\ldots ,{\tilde{s}}_{k}^{\left(J\right)}{]}^{T}$, ${\tilde{ı}}_{k}≜[{\tilde{ı}}_{k}^{\left(1\right)},\ldots ,{\tilde{ı}}_{k}^{\left(J\right)}{]}^{T}$, ${\tilde{\beta }}_{k}≜[{\tilde{\beta }}_{k}^{\left(1\right)},\ldots ,{\tilde{\beta }}_{k}^{\left(J\right)}{]}^{T}$, and ${\tilde{\gamma }}_{k}≜[{\tilde{\gamma }}_{k}^{\left(1\right)},\ldots ,{\tilde{\gamma }}_{k}^{\left(J\right)}{]}^{T}$, the mean of the epidemic states and model parameters at any time step *k*′ ∈ {*k*+1, … , *k* + *K*} are calculated as (cf. (14)) ${\hat{s}}_{k^{\prime }}^{F}≜J^{-1}{\tilde{s}}_{k^{\prime }}^{T}\cdot 1_{J}$, ${\hat{ı}}_{k^{\prime }}^{F}≜J^{-1}{\tilde{ı}}_{k^{\prime }}^{T}\cdot 1_{J}$, ${\hat{\beta }}_{k^{\prime }}^{F}≜J^{-1}{\tilde{\beta }}_{k^{\prime }}^{T}\cdot 1_{J}$, and ${\hat{\gamma }}_{k^{\prime }}^{F}≜J^{-1}{\tilde{\gamma }}_{k^{\prime }}^{T}\cdot 1_{J}$.

The steps of the proposed forecasting algorithm with stochastic SIR model are detailed in Algorithm 2.

## V. FORECAST PERFORMANCE ANALYSIS: SYNTHETIC DATA AND REAL COVID-19 OUTBREAK DATA

We present numerical results obtained with the sequential estimation and forecasting algorithm described in Section IV. In Section V-A the algorithm is applied to synthetic data, while real data from the recent COVID-19 outbreak are considered in Section V-B.

### A. SYNTHETIC DATA EXPERIMENT

**FIGURE 1.**
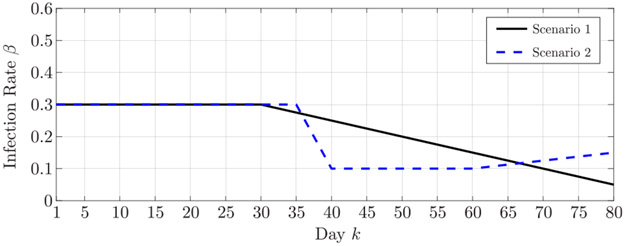
Evolution of the infection rate in the simulated scenarios.

The effectiveness of the proposed algorithm is validated in two simulated epidemic scenarios involving a community of *P* = 10^6^ individuals. The simulations span 80 days, during which the infection rate changes as shown in Fig. 1. The variations of the infection rate model the effects of the restriction measures established by the authorities: in the first scenario, the epidemic outbreak is controlled by long-term soft restriction measures that cause a slow, yet consistent, decrease in the infection rate; in the second scenario, an initial strict lockdown is then followed by a relaxation of the restriction measures that leads to a slight increase in the infection rate. The recovery rate is fixed and set to *γ* = 0.1. The initial state of the epidemic is described by the normalized numbers of susceptible, infected, and recovered individuals at time *k* = 0, that are *s*_0_ = 1 – *i*_0_ – *r*_0_, *i*_0_ = 20/P, and *r*_0_ = 1/*P*. The infection rate is discretized with *D*_1_ = 25 values between $\vartheta_{1}^{\left(1\right)}=0$ and $\vartheta_{D_{1}}^{\left(1\right)}=0.6$; the recovery rate with *D*_2_ = 10 values between $\vartheta_{1}^{\left(2\right)}=0$ and $\vartheta_{D_{2}}^{\left(2\right)}=0.2$. The prior pmf of the infection rate has mean ${\bar{\beta }}_{0}=0.4$ in the first scenario, and ${\bar{\beta }}_{0}=0.3$ in the second scenario; the standard deviation is *σ*_*β*_ = 0.1 for both the scenarios. Mean and standard deviation of the prior pmf of the recovery rate are ${\bar{\gamma }}_{0}=0.1$ and *σ*_*γ*_ = 0.04, respectively, for both the scenarios. The transition matrix for the parameter *β*_*k*_ defined in (35) is set to [**P**_*β*_]_ℓ,ℓ_ = 0.9 for ℓ ∈ {1, … , *D*_1_}, [**P**_*β*_]_ℓ–1,ℓ_ = [**P**_*β*_]_ℓ+1,ℓ_ = 0.05 for ℓ ∈ {2, … , *D*_1_ – 1}, and [**P**_*β*_]_2,1_ = [**P**_*β*_]_*D*_1_–1,*D*_1__ = 0.1. The transition matrix for the parameter *γ*_*k*_ defined in (36) is analogously set to [**P**_*γ*_]_ℓ,ℓ_ = 0.99 for ℓ ∈ {1, … , *D*_2_}, [**P**_*γ*_]_ℓ–1,ℓ_ = [**P**_*γ*_]_ℓ+1,ℓ_ = 0.005 for ℓ ∈ {2, … , *D*_2_ – 1}, and [**P**_*γ*_]_2,1_ = [**P**_*γ*_]_*D*_2_–1,*D*_2__ = 0.01. The number of mixture components is *N* = 5, and mean and covariance matrix of the prior pdf of the *n*^th^ component are

$${\bar{x}}_{0}^{\left(n\right)}=\left[\begin{array}{c} s_{0}\\ i_{0} \end{array}\right]+\left[\begin{array}{cc} -1 & -1\\ 1 & 0 \end{array}\right]\left[\begin{array}{c} \varepsilon_{1}^{\left(n\right)}\\ \varepsilon_{2}^{\left(n\right)} \end{array}\right]$$

and ${\bar{C}}_{0}^{\left(n\right)}\;=\;{\bar{C}}_{0}\;=\;I_{2}i_{0}$, respectively, where $\varepsilon_{1}^{\left(n\right)}\;∼\;U(-i_{0}∕5,i_{0}∕5)$ and $\varepsilon_{2}^{\left(n\right)}\;∼\;U(-r_{0}∕5,r_{0}∕5)$. Finally, the observation noise covariance matrix in (28) is **R**_c_ = 50 **I**_2_. As for the forecasting, the ensemble size is *J* = 2 × 10^4^, and the minimum and maximum numbers of points used to estimate the slope of the infection rate are *L*_min_ = 5 and *L*_max_ = 14, respectively.

**FIGURE 2.**
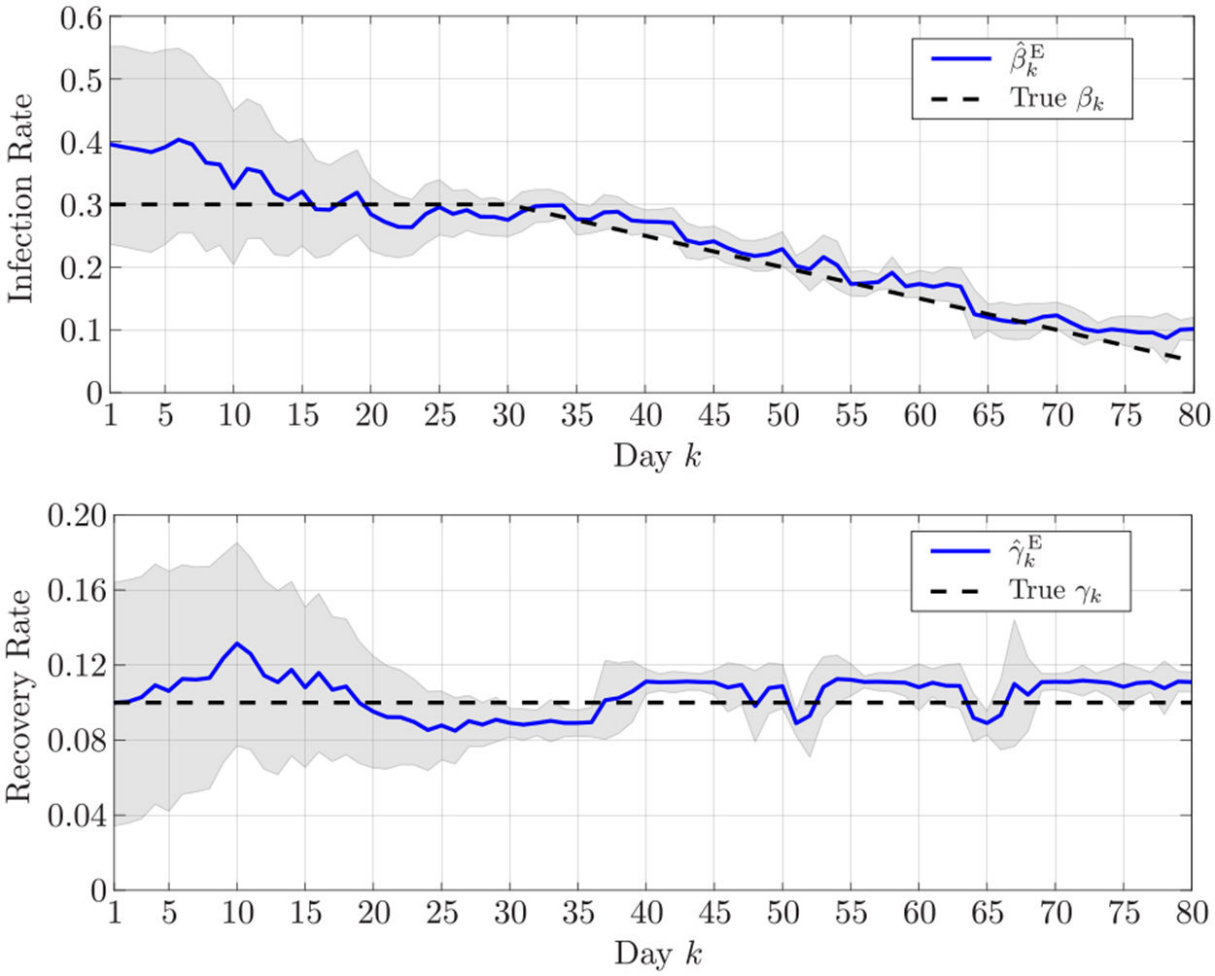
Estimated (top) infection rate and (bottom) recovery rate in the first simulated scenario. The shaded areas represent the 90% confidence interval.

**FIGURE 3.**
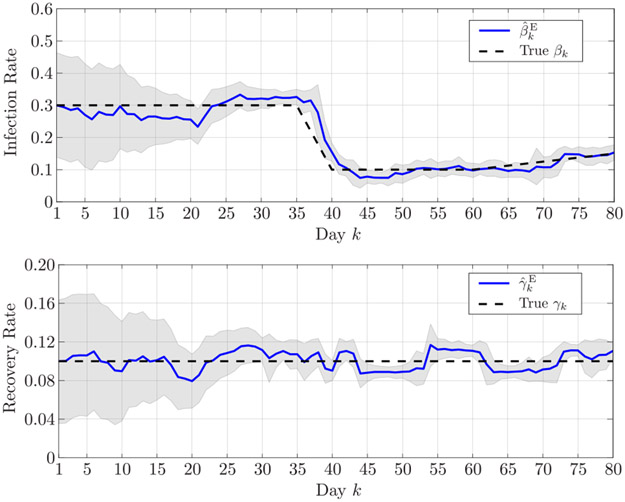
Estimated (top) infection rate and (bottom) recovery rate in the second simulated scenario. The shaded areas represent the 90% confidence interval.

Fig. 2 shows the infection and recovery rates estimated in the first scenario over the 80 days, along with their 90% confidence intervals. Analogously, Fig. 3 shows the estimated infection and recovery rates in the second scenario. The results demonstrate the capability of proposed Bayesian sequential estimation algorithm to closely follow the time variation of the infection rates even in the presence of abrupt fluctuations, as well as to accurately estimate the recovery rate.

**FIGURE 4.**
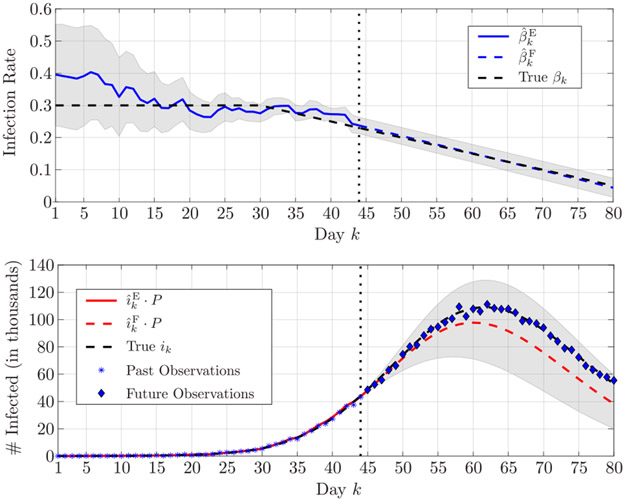
Estimation and forecasting, respectively in solid and dashed lines, of (top) the infection rate and (bottom) the number of infected individuals in the first scenario; the superscripts E and F stand for *estimate* and *forecast*, respectively. The estimation is up to *k* = 44 (marked by a vertical dotted line), and the forecast is up to *k* = 80. The shaded areas represent the 90% confidence interval.

**FIGURE 5.**
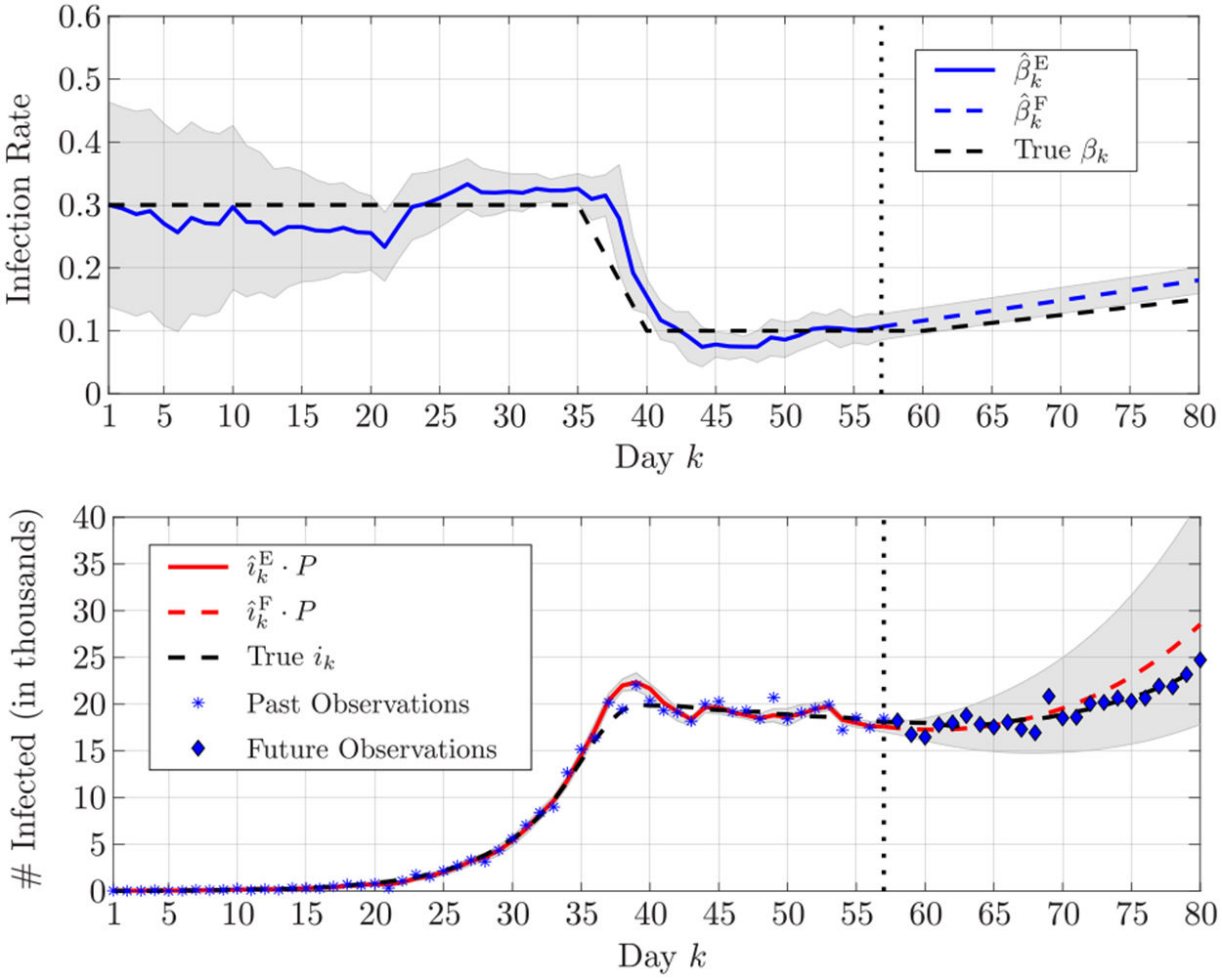
Estimation and forecasting, respectively in solid and dashed lines, of (top) the infection rate and (bottom) the number of infected individuals in the second scenario; the superscripts E and F stand for *estimate* and *forecast*, respectively. The estimation is up to *k* = 57 (marked by a vertical dotted line), and the forecast is up to *k* = 80. The shaded areas represent the 90% confidence interval.

In turn, the accuracy of the proposed algorithm allows one to reliably forecast the epidemic evolution. Fig. 4 presents the estimation and forecast on the infection rate and of the number of infected in the first scenario; we assume that the latest available observation is on day *k* = 44 — so that the estimation stops on this day —, and the forecast is up to day *k* = 80. The forecast of the number of infected individuals well represents the evolution of the epidemic, suggesting a peak between days 55 and 65. Furthermore, we observe how both the true infection rate and the true number of infected is always enveloped within the 90% confidence interval, showing the high reliability of the proposed algorithm. Finally, in Fig. 5, we show the forecast of the epidemic evolution in the second scenario. Here, the estimation is performed up to day *k* = 57; the capability of the proposed algorithm to accurately estimate the large variation in the infection rate and forecast its future average value, allows one to forecast the evolution of the number infected, even though a further small variation of the infection rate will start at *k* = 60.

### B. REAL DATA: COVID-19 OUTBREAK

This section presents the results obtained with the proposed estimation and forecasting algorithm when applied to real data obtained from the recent COVID-19 outbreak. The focus is on two very different areas in terms of population and interactions: Lombardia region in Italy, and the USA.

#### 1) LOMBARDIA REGION, ITALY

**FIGURE 6.**
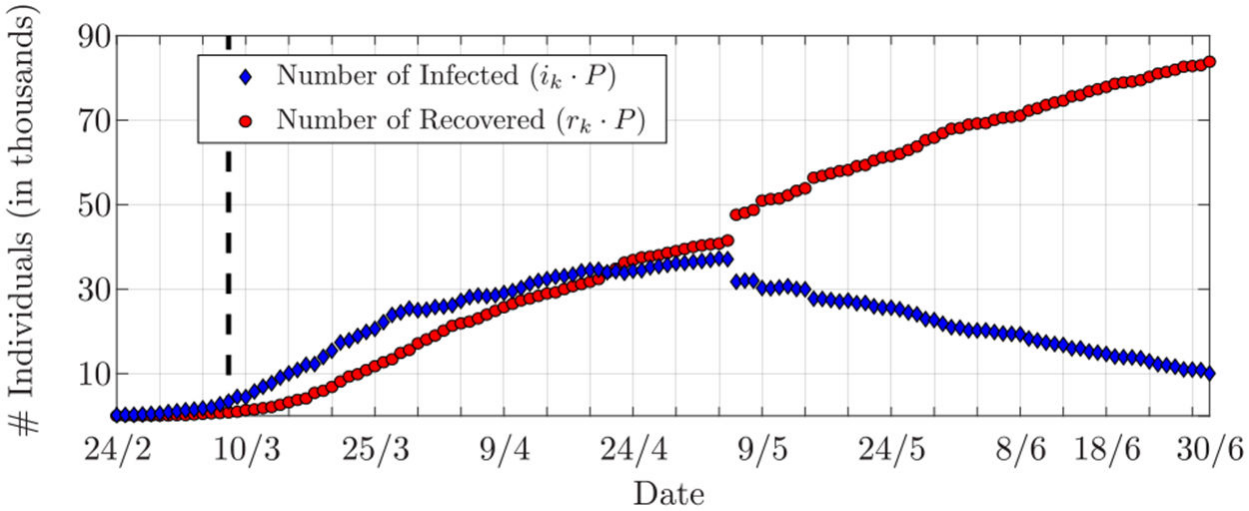
Numbers of infected and recovered (i.e., hospital releases plus deaths) individuals in Lombardia, Italy, from February 24, 2020, to June 30, 2020 (data from Protezione Civile [38]). The vertical dashed line indicates March 8, 2020, the beginning of the lockdown. The large steps on May 6 are due to an inaccurate reporting of the data, as explained in Section V-B1.

Official data on the COVID-19 epidemic outbreak in Italy are made available from Protezione Civile on a daily basis [38]. This includes many entries, both nationwide and per region, as the total number of cases, total number of current positive cases, new positive cases per day, number of hospitalised patients, number of tests performed, number of discharged COVID-19 patients from the hospitals, and number of deaths. Here, we focus on the data from Lombardia region, the centre of Italy’s COVID-19 outbreak, whose population is *P* ≈ 10^7^ people. We used the normalized (to *P*) total number of current positive cases as number of infected *i*_*k*_, and the normalized sum of number of discharged patients and number of deaths as the number of recovered *r*_*k*_. These are reported in Fig. 6 and refer to the period between February 24, 2020, and June 30, 2020. The figure also shows the beginning of the lockdown established by the Italian government on March 8, 2020. Furthermore, we observe that, on May 6, the number of infected and number of recovered individuals present large steps, which hardly reflect physical reality. These steps are due to the fact that the numbers reported on May 6 include not only data referring to that day, but also data collected on previous days, and, erroneously, not reported in the correct day [38].

The setting of the Bayesian sequential estimation and forecasting algorithm is as described in Section V-A, except that the smallest and largest values used for the discretization of the infection rate are $\vartheta_{1}^{\left(1\right)}=0$ and $\vartheta_{D_{1}}^{\left(1\right)}=0.4$, respectively; the smallest and largest values used for the discretization of the recovery rate are $\vartheta_{1}^{\left(2\right)}=0$ and $\vartheta_{D_{2}}^{\left(2\right)}=0.1$, respectively; mean and standard deviation of their prior pmfs are ${\bar{\beta }}_{0}=0.3$ and *σ*_*β*_ = 0.07, and ${\bar{\gamma }}_{0}=0.06$ and *σ*_*γ*_ = 0.02; and the observation noise covariance matrix is **R**_c_ = 100 **I**_2_. The initial state of the epidemic is given by the normalized numbers of susceptible, infected, and recovered on February 24, that are *s*_0_ = 1 – *i*_0_ – *r*_0_, *i*_0_ = 166/*P*, and *r*_0_ = 6/*P*.

**FIGURE 7.**
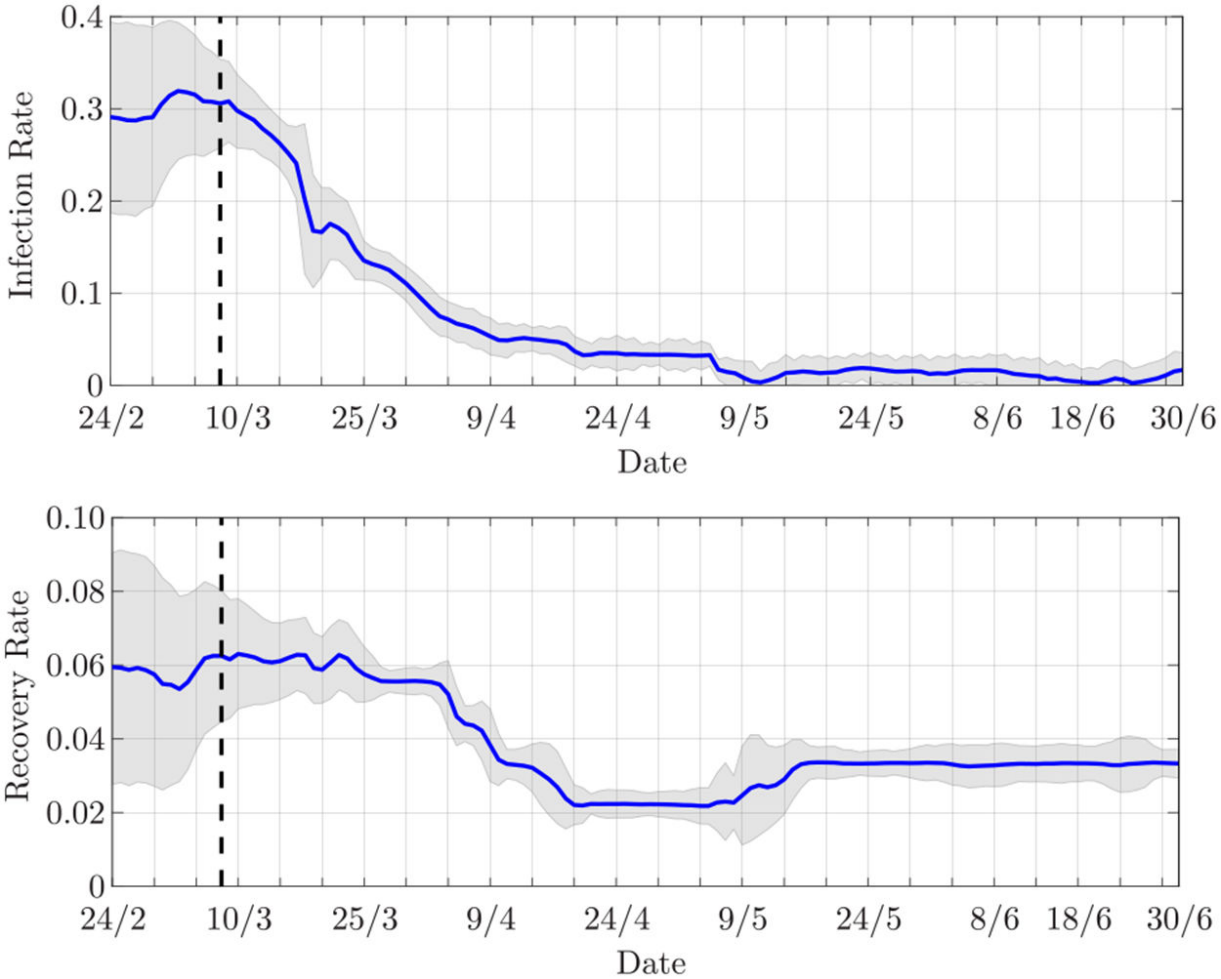
Estimated (top) infection rate and (bottom) recovery rate for Lombardia. The vertical dashed line indicates March 8, 2020, the beginning of the lockdown. The shaded areas represent the 90% confidence interval.

The estimated infection and recovery rates are shown in Fig. 7. The decrease in the infection rate, which represents the slowdown of the epidemic, clearly reflects the restriction measures established on March 8. The recovery rate, instead, decreases up to May 6, when it then shows a slight increase. The reduction of the recovery rate balances the decrease in the infection rate; indeed, up to May 6, the number of infected is still growing, which suggests that the infection rate is greater than the recovery rate, i.e., *β* > *γ*. After May 6, this trend changes.

**FIGURE 8.**
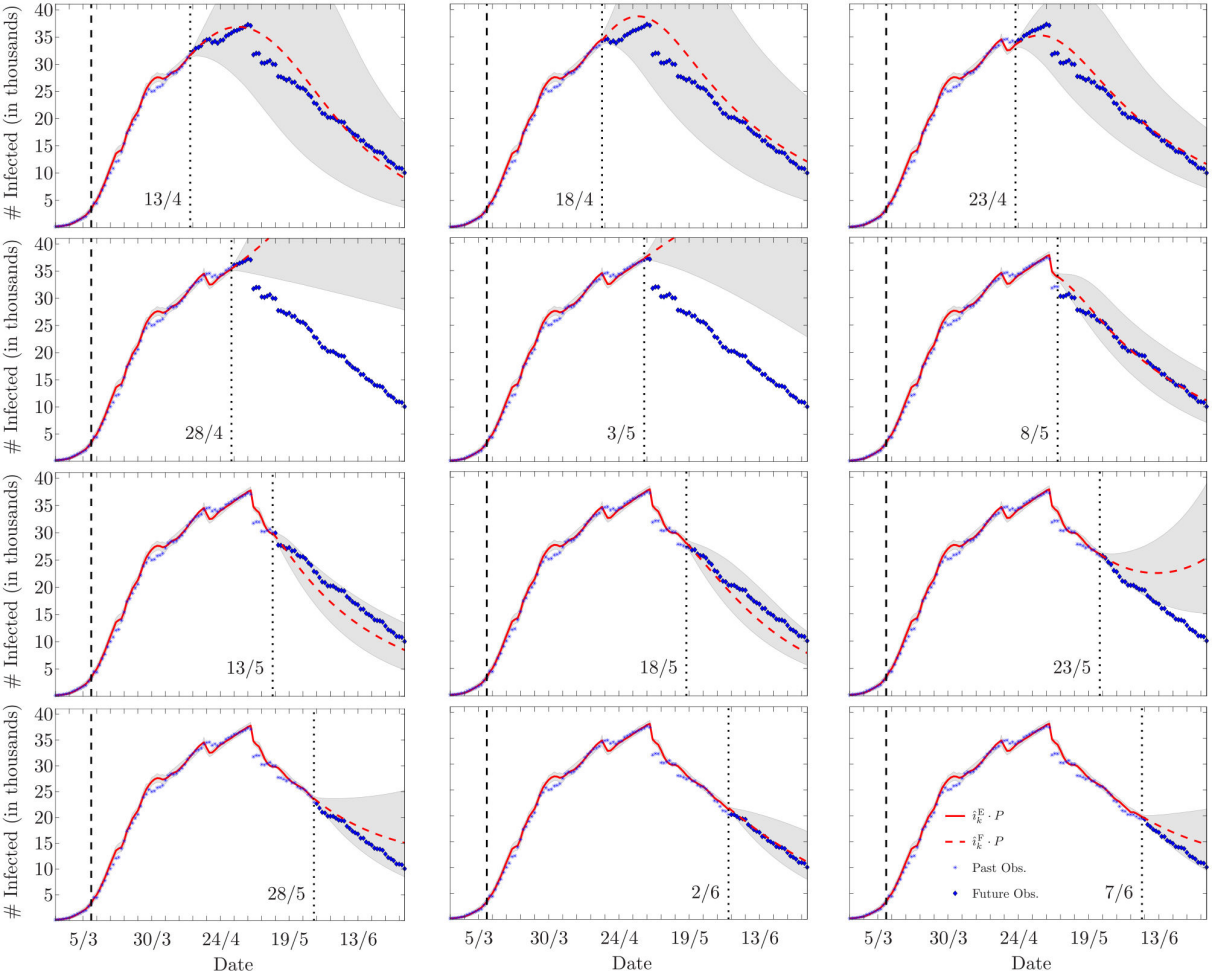
Estimation and forecasting, respectively in solid and dashed lines, of the number of infected individuals in Lombardia, Italy (legend is reported in the bottom-right corner image; the superscript E stands for *estimate*, and the superscript F stands for *forecast*). The date corresponding to the end of the estimation and the beginning of the forecast is marked by a vertical dotted line (the leftmost vertical dashed line marks March 8, the beginning of the lockdown). In all the cases, the forecast horizon is June 30. The shaded area represents the 90% confidence interval. The poor forecasts made on April 28, and May 3, relate to the inaccurate data later provided on May 6, as explained in Section V-B1.

Fig. 8 reports the forecasts of the epidemic evolution assessed every five days in the time period between April 13 and June 7, and Table 1 presents the mean absolute percentage errors (MAPEs) calculated for each forecast and for different forecast horizons, that is, 3, 7, and 14 days. We note that the forecasts made on April 13, 18, and 23, follow the future observations well, with an average MAPE below 3% at a forecast horizon of 7 days. On April 28 and May 3 the forecasts are not reliable, since the future observations are not contained within the 90% confidence interval. However, this poor performance is related to the inaccurate data provided later on May 6; indeed, the next forecasts made from May 8, to June 7, present again low MAPEs, with an average of 3.49 %, 4.24 %, and 6.1 %, at forecast horizons of 3, 7, and 14 days, respectively. Neglecting the forecasts whose horizon includes May 6 (marked with an asterisk in Table 1), the average MAPE from April 13, to June 7, is 3.6% for forecasts at 7 days, and below 6% when the forecast horizon is 14 days.

The proposed algorithm is compared with two alternative curve-fitting approaches. The first one, hereafter named SIR-fit, employs a nonlinear least squares fitting algorithm that, using the number of infected and recovered individuals, computes the *best*^6^ time-invariant infection and recovery rates of the deterministic SIR model. These *best* rates are then used to forecast the evolution of the epidemic. The second curve-fitting approach follows the same methodology applied on a more-sophisticated recently proposed generalized SEIR (GSEIR) model [39], for this reason hereafter called GSEIR-fit. The GSEIR model consists of seven compartments — three more compartments than those in the standard SEIR model, i.e., insusceptible, quarantined, and death — and six parameters. Table 2 compares the average MAPEs obtained with the proposed algorithm, the SIR-fit, and the GSEIR-fit. The comparison is made averaging the MAPEs over two different time intervals. The first interval is from March 4, i.e., the 10th day since the beginning of the data collection, to June 16; the second interval is from April 1, i.e., approximately three weeks after the lockdown, to June 16. The proposed algorithm clearly outperforms the SIR-fit for all the forecast horizons. The GSEIR-fit, instead, presents a single lower average MAPE over the interval from March 4 to June 16 when the forecast horizon is 14 days; however, when the interval from April 1 to June 16 is considered, the proposed algorithm outperforms the GSEIR-fit in all the cases. This confirms the benefit of sequentially estimating the time-varying model parameters, in order to have reliable and accurate forecasts.

#### 2) UNITED STATES OF AMERICA

**FIGURE 9.**
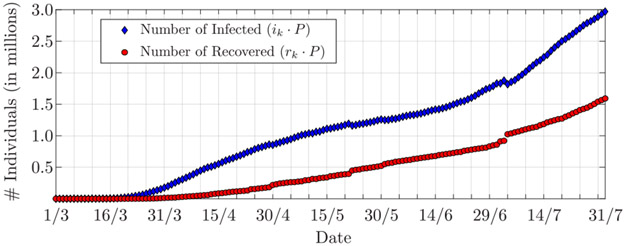
Numbers of infected and recovered (i.e., hospital releases plus deaths) individuals in the USA, from March 1, 2020, to July 31, 2020 (data from JHU [41]).

Since the beginning of the COVID-19 epidemic outbreak, the Johns Hopkins University (JHU) has tracked the evolution of the contagion and made the collected data publicly available [40], [41]. The repository includes the total number of cases, the number of deaths, and the number of discharged COVID-19 patients from the hospitals from the USA and other countries at different levels of details, i.e., for the country as a whole and, when available, for single states and regions. Here, we use the overall dataset from the USA, whose population is *P* ≈ 329.8 · 10^6^ people. As for the experiment made on the dataset from Lombardia, the normalized (to *P*) sum of number of discharged patients and number of deaths is used as the number of recovered *r*_*k*_; the normalized number of infected *i*_*k*_ is then given by the normalized difference between the total number of cases and the number of recovered. These are reported in Fig. 9 and refer to the period between March 1, 2020, and July 31, 2020.

The setting of the Bayesian sequential estimation and forecasting algorithm is unchanged, except that for the discretization and initialization of the parameters, and the initialization of the epidemic state. Specifically, the smallest and largest values used for the discretization of the infection rate are $\vartheta_{1}^{\left(1\right)}=0$ and $\vartheta_{D_{1}}^{\left(1\right)}=0.5$, respectively; the smallest and largest values used for the discretization of the recovery rate are $\vartheta_{1}^{\left(2\right)}=0$ and $\vartheta_{D_{2}}^{\left(2\right)}=0.05$, respectively; mean and standard deviation of their prior pmfs are ${\bar{\beta }}_{0}=0.35$ and *σ*_*β*_ = 0.08, and ${\bar{\gamma }}_{0}=0.015$ and *σ*_*γ*_ = 0.008; and the observation noise covariance matrix is **R**_c_ = 2000 **I**_2_. The initial state of the epidemic is given by the normalized numbers of susceptible, infected, and recovered on March 1, that are *s*_0_ = 1 – *i*_0_ – *r*_0_, *i*_0_ = 22/*P*, and *r*_0_ = 8/*P*.

**FIGURE 10.**
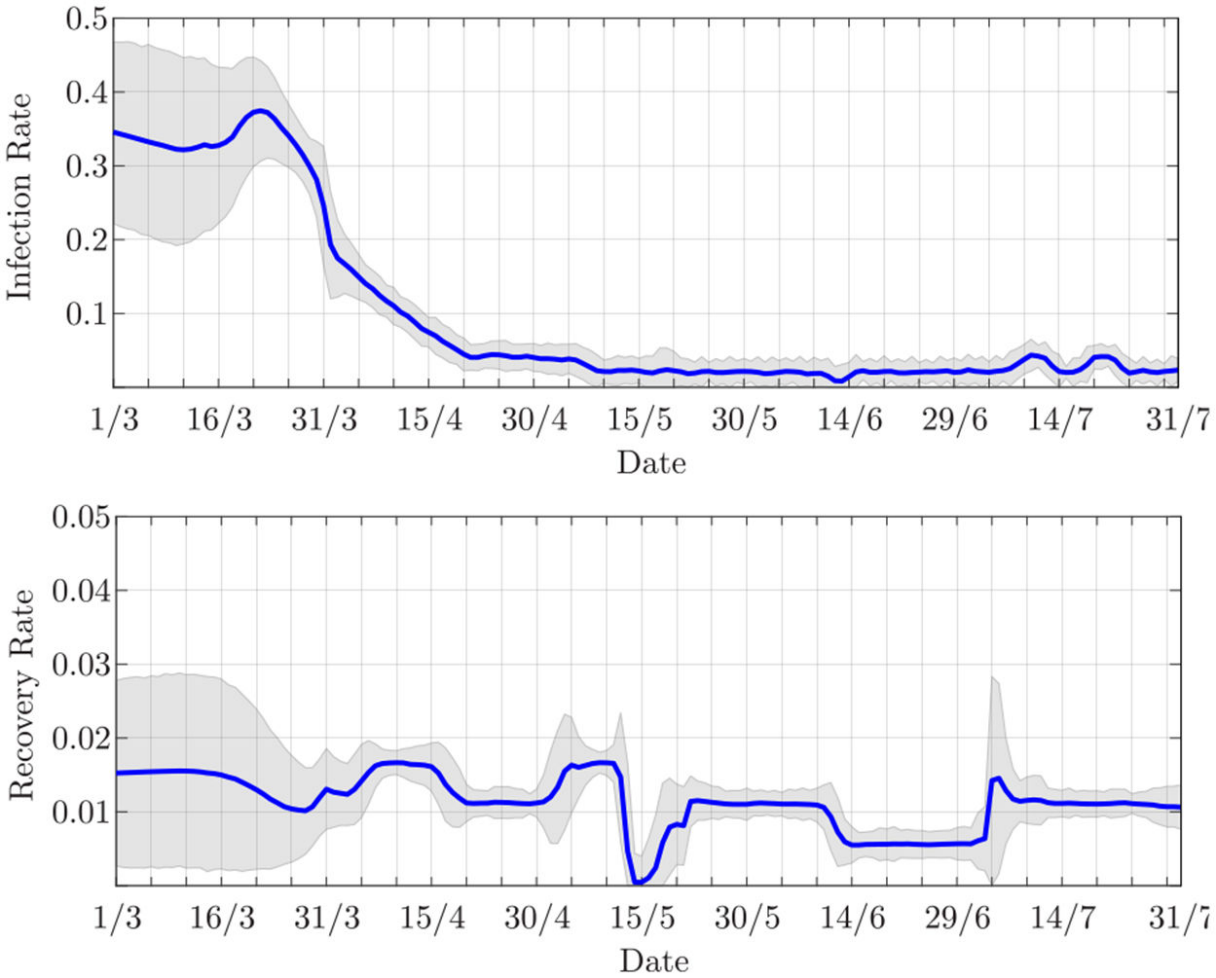
Estimated (top) infection rate and (bottom) recovery rate for the USA. The shaded areas represent the 90% confidence interval.

Fig. 10 shows the estimated infection and recovery rates. From the second half of March and through April the infection rate decreases, presumably due to the restriction measures established by each single State. Here, we cannot mark a specific date as the beginning of the lockdown; nevertheless, it is reasonable to assume that about three out of four US citizens were under some form of lockdown by early April [42]. Around April 30, the estimated recovery rate shows a slight increase followed by an abrupt decrease. On that day, 35 thousand new recovered (i.e., hospital releases plus deaths) individuals were reported against a decrease of infected individuals of only 6 thousand (cf. Fig. 9); this results in a sudden increase of the recovery rate. Large numbers of new recovered individuals are also reported on May 22 and July 4, that are, 53 thousand and 104 thousand, respectively; however, these are better balanced by the numbers of people leaving the infected group, that are, 29 thousand and 58 thousand, respectively, thus not significantly affecting the estimates of the infection and recovery rates.

Overall, the estimated recovery rate is roughly 0.01, which translates into an average number of 100 days that an individual takes to move from the group of infected (I) to the group of recovered (R). Although the recovery duration seems overestimated, it is worth highlighting that this is an aggregate estimate of the recovery rate from multiple States, which therefore suffers from multiple different reporting delays, as well as from the different criteria used to declare an individual as fully recovered. It underscores the need for the USA to provide timely and consistent data, similar to that just analysed in Lombardia, if public health policy is to be driven by reliable estimation and prediction.

**FIGURE 11.**
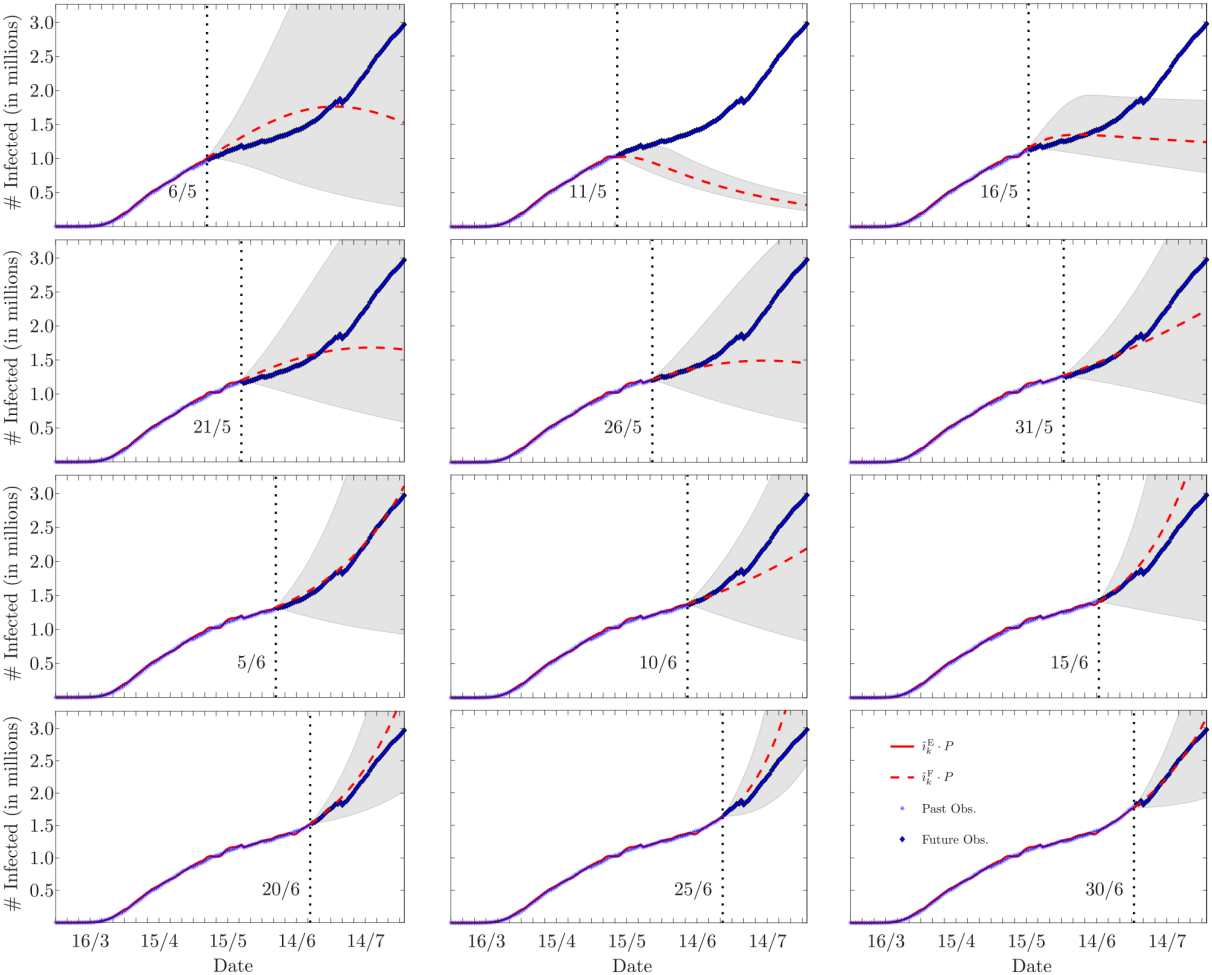
Estimation and forecasting, respectively in solid and dashed lines, of the number of infected individuals in the USA (legend is reported in the bottom-right corner image; the superscript E stands for *estimate*, and the superscript F stands for *forecast*). The date corresponding to the end of the estimation and the beginning of the forecast is marked by a vertical dotted line. In all the cases, the forecast horizon is July 31. The shaded area represents the 90% confidence interval. The poor forecasts made on May 11 and 16, relate to the abrupt decrease of the estimated recovery rate that follows April 30 (cf. Fig. 10, bottom image).

Forecasts of the epidemic evolution evaluated every five days in the time period between May 6 and June 30 are reported in Fig. 11. Table 3 presents the MAPEs calculated for each forecast and for different forecast horizons, that is, 3, 7, and 14 days.

We note that the forecasts made on May 6, 11, 16, and 21, present the highest MAPEs; these forecasts, however, are negatively affected by the abrupt decrease of the recovery rate that follows April 30, as described above. The forecasts made from May 26, to June 30, instead, present MAPEs that are always below 3% at forecast horizons of 3 and 7 days, and always below 4% when the forecast horizon is 14 days. Overall, the results in Table 3 confirm those obtained with the data from Lombardia: the average MAPE for forecast horizons of 3, 7, and 14 days, is, respectively, 2.35 %, 3.03 %, and 4.16 %. Table 4 compares the average MAPEs obtained with the proposed algorithm, with those obtained with the SIR-fit and the GSEIR-fit curve-fitting approaches. The proposed algorithm consistently outperforms both the curve-fitting approaches, in both the considered time intervals. Moreover, we observe a significant improvement of the MAPE for the proposed algorithm when considering the interval from April 1 to July 17, compared to the interval from March 10 to July 17, which again confirms the benefit of sequentially estimating the time-varying model parameters in pursuance of reliable and accurate forecasts.

### C. LIMITATIONS AND EXTENSIONS

The proposed analysis presents some limitations that may lead to future extensions and these are worth exploring. First, the considered stochastic SIR model may be broadened to include the fraction of undocumented (or asymptomatic) and quarantined infected individuals. Undocumented infections usually include mild or asymptomatic cases that go undetected and, hence, based on their proportion and contagiousness, can potentially increase the spread of the disease. The portion of undocumented infectious cases is suspected to be a critical epidemiological characteristic that is not easy to quantify. Most of the available evidence on asymptomatic SARS-CoV-2 infections, reviewed and summarised in [43] for different circumscribed cohorts, suggests that this is a significant factor in the fast progression of the COVID-19 pandemic. However, the difficulty in quantification of undocumented cases is largely due to the imperfection of the data available, which does not accurately reflect a large, representative sample of the general population. Moreover, in order to distinguish asymptomatic and presymptomatic cases, longitudinal data — that is, repeated observations of the individuals over time — should be available.

Another possible extension may be to separate people who are confirmed infected and home-quarantined into a dedicated epidemic compartment. In addition, the recovered compartment typical of the SIR model may be separated into two distinct recovery and death compartments in the detection phase so that the available data on reported cases can be taken into account separately. Furthermore, the considered stochastic SIR model describes the spread of the disease inside a single and confined population, e.g., a city, a region (Lombardia, Italy), a nation (USA). However, the approach presented in this article could be extended to more complicated metapopulation models, which introduce a further spatial travel/diffusion dimension in the dynamic and observation models. In particular, the population could be represented as a network of multiple spatially separated subpopulations nodes, such as multiple cities in the same region or nation. The interconnections among different populations represents the diffusion of people, thus contributing to the disease spread among the different subpopulations. All these limitations can be addressed in future studies because they mainly affect data analysis, and do not restrict the application and the effectiveness of the proposed approach to learning and forecasting the evolution of the critical epidemiological characteristics.

## VI. CONCLUSIONS

The recent worldwide epidemic outbreak, due to a new strain of Coronavirus, has intensified research into novel mathematical models and algorithms that are able to reliably estimate and predict the epidemiological curve of the infection. In this article, we proposed a Bayesian sequential estimation and forecasting algorithm that, based on the information that authorities provide on a daily basis, is able to estimate the state of the epidemic and the parameters of the underlying model, as well as to forecast the evolution of the epidemiological curve. We developed an efficient implementation of the above-mentioned Bayesian framework, specifically tailored to the stochastic SIR model of pandemic evolution. The proposed algorithm is validated using synthetic data simulating two epidemic scenarios, and on real data acquired during the recent COVID-19 outbreak in the Lombardia region, Italy, and in the USA. Results show that the mean absolute percentage error computed after the lockdown is on average below 5% when the forecast is at 7 days, and below 10% when the forecast horizon is 14 days. Moreover, the described Bayesian framework outperforms curve-fitting approaches that use deterministic epidemiological models (e.g., SIR and GSEIR), particularly when a clear change of model parameters occur, e.g., a decrease of the infection rate following the lockdown. Finally, accurate and timely data collection, especially on recovered individuals, hospitalizations, intensive care unit admissions, and intubations, is essential for reliable model-based decisions.

There exists an enormous amount of very recent literature related to the forecast of COVID-19 pandemic evolution, part of which has been reviewed at the beginning of this article. The analysis of this literature makes clear the effectiveness of model-based approaches, over less structured data-centric methodologies. In this respect, one lesson learned by the present study is that accurate epidemic modeling requires accurate estimation of time-varying parameters, such as the infection rate *β*. This is obviously true in the presence of abrupt changes of the underlying physical situation (e.g., adoption of drastic countermeasures) but, more interestingly, it is by no means limited to these extreme situations. One consequence is that, once the epidemic is under control, small variations in the estimated *β* may be used as a sensible proxy for the incipient detection of possible pandemic recrudescence.

## Figures and Tables

**TABLE 1.** Mean absolute percentage errors (MAPEs) of the forecasts of the epidemic evolution in Lombardia, Italy, performed at different dates and calculated for different forecast horizons, that is, 3, 7, and 14 days. The asterisk means that the forecast performed at a given date and with a given forecast horizon includes May 6, when inaccurate numbers of infected and recovered individuals were reported. The average (last row) does not take into account these cases.

Forecast Date	3 Days (%)	7 Days (%)	14 Days (%)
April 13	0.54	0.50	2.49
April 18	3.47	6.48	6.84
April 23	1.25	1.91	3.88*
April 28	0.42	1.07	14.91*
May 3	8.02*	18.44*	30.07*
May 8	9.14	7.47	6.31
May 13	2.30	2.59	6.48
May 18	0.92	2.24	3.29
May 23	0.69	3.78	9.02
May 28	4.67	5.10	4.99
June 2	2.70	1.87	1.93
June 7	4.02	6.61	10.76
Average	2.74	3.60	5.79

**TABLE 2.** Average mean absolute percentage errors (MAPEs) of the forecasts of the epidemic evolution in Lombardia, Italy, obtained with the proposed algorithm, and with the SIR-fit and GSEIR-fit curve-fitting approaches, for different forecast horizons, that is, 3, 7, and 14 days. The uppermost table reports the average MAPEs computed over the interval from March 4 to June 16; the lowermost table reports the average MAPEs computed over the interval from April 1 to June 16.

Algorithm	3 Days (%)	7 Days (%)	14 Days (%)
Proposed	6.2	10.4	23.5
SIR-fit	77.5	115.3	225.8
GSEIR-fit	10.3	13.0	18.5
Average over the interval from March 4 to June 16			
Algorithm	3 Days (%)	7 Days (%)	14 Days (%)
Proposed	3.3	4.9	9.4
SIR-fit	88.2	123.0	213.2
GSEIR-fit	11.6	13.6	16.8
Average over the interval from April 1 to June 16			

**TABLE 3.** Mean absolute percentage errors (MAPEs) of the forecasts of the epidemic evolution in the USA, performed at different dates and calculated for different forecast horizons, that is, 3, 7, and 14 days.

Forecast Date	3 Days (%)	7 Days (%)	14 Days (%)
May 6	4.31	5.73	7.44
May 11	4.55	7.25	11.80
May 16	4.84	5.97	6.87
May 21	5.01	5.61	6.71
May 26	0.61	0.98	0.96
May 31	2.58	2.58	2.87
June 5	2.29	2.86	3.49
June 10	1.09	0.90	1.25
June 15	0.26	0.70	1.51
June 20	1.15	1.04	1.52
June 25	0.40	0.49	3.93
June 30	1.11	2.28	1.52
Average	2.35	3.03	4.16

**TABLE 4.** Average mean absolute percentage errors (MAPEs) of the forecasts of the epidemic evolution in the USA, obtained with the proposed algorithm, and with the SIR-fit and GSEIR-fit curve-fitting approaches, for different forecast horizons, that is, 3, 7, and 14 days. The uppermost table reports the average MAPEs computed over the interval from March 10 to July 17; the lowermost table reports the average MAPEs computed over the interval from April 1 to July 17.

Algorithm	3 Days (%)	7 Days (%)	14 Days (%)
Proposed	7.3	11.9	29.8
SIR-fit	81.6	122.4	232.4
GSEIR-fit	14.0	20.0	35.2
Average over the interval from March 10 to July 17			
Algorithm	3 Days (%)	7 Days (%)	14 Days (%)
Proposed	3.4	4.5	7.2
SIR-fit	91.4	138.8	267.6
GSEIR-fit	12.1	17.0	28.7
Average over the interval from April 1 to July 17			

## Footnotes

1 At the time of writing, permanent immunity of recovered COVID-19 patients is uncertain. However, it is a bedrock feature of the SIR epidemiological model.

2 This applies, e.g., to the influenza virus, as its infection rate periodically increases and decreases depending on the season.

3 The superscript E stands for *estimate*, and is used to distinguish the estimate from the *forecast*, later identified by the superscript F.

4 By “evolving” is meant a simple procedure of recursively (in time) generating random variables according to a transition distribution; colloquially, this is a process of “rolling the dice”.

5 Note that authors generally refer to the population size as *N* [4], [5], [7]-[13].

6 *Best* is meant in the least-squares sense.

## APPENDIX A

### MOMENT MATCHING

This section reports the expressions, derived by moment matching, used to compute ${\hat{x}}_{k+1|k}^{\left(n,\theta_{k}\right)}$ and ${\hat{C}}_{k+1|k}^{\left(n,\theta_{k}\right)}$ in (34). Let ${\hat{x}}_{k|k}^{\left(n,\theta_{k}\right)}≜[{\hat{s}}_{k|k}^{\left(n,\theta_{k}\right)},{\hat{ı}}_{k|k}^{\left(n,\theta_{k}\right)}{]}^{T}$ and

$${\hat{C}}_{k|k}^{\left(n,\theta_{k}\right)}≜\left[\begin{array}{cc} {\left(\Sigma_{s,k}^{\left(n\right)}\right)}^{2} & \rho_{k}^{\left(n\right)}\Sigma_{s,k}^{\left(n\right)}\Sigma_{i,k}^{\left(n\right)}\\ \rho_{k}^{\left(n\right)}\Sigma_{s,k}^{\left(n\right)}\Sigma_{i,k}^{\left(n\right)} & {\left(\Sigma_{i,k}^{\left(n\right)}\right)}^{2} \end{array}\right]$$

be mean and covariance matrix of the *n*^th^ posterior mixand $P(x_{k}|n,\theta_{k},z_{1:k})$. We aim to approximate the *n*^th^ predicted mixand $P(x_{k+1}|n,\theta_{k},z_{1:k})$ to be Gaussian with mean

$${\hat{x}}_{k+1|k}^{\left(n,\theta_{k}\right)}≜E\left[x_{k+1}|n,\theta_{k},z_{1:k}\right]={\left[{\hat{s}}_{k+1|k}^{\left(n,\theta_{k}\right)},{\hat{ı}}_{k+1|k}^{\left(n,\theta_{k}\right)}\right]}^{T}$$

and covariance matrix

$${\hat{C}}_{k+1|k}^{\left(n,\theta_{k}\right)}\;\;≜E\left[(x_{k+1}-{\hat{x}}_{k+1|k})\;(x_{k+1}-{\hat{x}}_{k+1|k}{)}^{T}|n,\theta_{k},z_{1:k}\right]\;\;=E\left[x_{k+1}x_{k+1}^{T}|n,\theta_{k},z_{1:k}\right]-{\hat{x}}_{k+1|k}{\hat{x}}_{k+1|k}^{T}\;\;=[\begin{array}{cc} {\left(\Sigma_{s,k+1}^{\left(n\right)}\right)}^{2} & \rho_{k+1}^{\left(n\right)}\Sigma_{s,k+1}^{\left(n\right)}\Sigma_{i,k+1}^{\left(n\right)}\\ \rho_{k+1}^{\left(n\right)}\Sigma_{s,k+1}^{\left(n\right)}\Sigma_{i,k+1}^{\left(n\right)} & {\left(\Sigma_{i,k+1}^{\left(n\right)}\right)}^{2} \end{array}].$$

To simplify the notation, we hereafter omit the index *n* and the dependence on the parameter vector ***θ***_*k*_. Following simple algebraic calculations, the components of the mean vector are obtained as

$${\hat{s}}_{k+1|k}=(1-\beta_{k}{\hat{ı}}_{k|k}\Delta_{t}){\hat{s}}_{k|k}-\beta_{k}\Delta_{t}\rho_{k}\Sigma_{s,k}\Sigma_{i,k},$$

and

$${\hat{ı}}_{k+1|k}=(1+\beta_{k}{\hat{s}}_{k|k}\Delta_{t}-\gamma_{k}\Delta_{t}){\hat{ı}}_{k|k}+\beta_{k}\Delta_{t}\rho_{k}\Sigma_{s,k}\Sigma_{i,k}.$$

We note that the products ${\hat{ı}}_{k|k}{\hat{s}}_{k|k}$ induce nonlinearity in their predicted values, and hence require extra calculation for the associated uncertainties; indeed, the components of the covariance matrix are

Σi,k+12=(1−γkΔt)2(ı^k∣k2+Σi,k2)+βk2Δt2[s^k∣k2ı^k∣k2+s^k∣k2Σi,k2+ı^k∣k2Σs,k2][+(1+2ρk2)Σs,k2Σi,k2+4ρks^k∣kı^k∣kΣs,kΣi,k]+2(1−γkΔt)βkΔt[s^k∣k(ı^k∣k2+Σi,k2)][+2ρkı^k∣kΣs,kΣi,k]+P−1Δt[βk(s^k∣kı^k∣k]+[ρkΣs,kΣi,k)+γkı^k∣k]−ı^k+1∣k2,

Σs,k+12=s^k∣k2+Σs,k2+βk2Δt2[s^k∣k2ı^k∣k2]+s^k∣k2Σi,k2+ı^k∣k2Σs,k2+(1+2ρk2)Σs,k2Σi,k2[+4ρks^k∣kı^k∣kΣs,kΣi,k,]−2βkΔt[ı^k∣k(s^k∣k2+Σs,k2)+2ρks^k∣kΣs,kΣi,k]+P−1βkΔt(s^k∣kı^k∣k+ρkΣs,kΣi,k)−s^k+1∣k2,

and

ρk+1Σs,k+1Σi,k+1=(1−γkΔt−P−1βkΔt)(s^k∣kı^k∣k+ρk,nΣs,kΣi,k)−(1−γkΔt)βkΔt[s^k∣k(ı^k∣k2+Σi,k2)][+2ρkı^k∣kΣs,kΣi,k]+βkΔt[ı^k∣k(s^k∣k2+Σs,k2)][+2ρks^k∣kΣs,kΣi,k]−βk2Δt2[s^k∣k2ı^k∣k2+s^k∣k2Σi,k2]+ı^k∣k2Σs,k2+(1+2ρk2)Σs,k2Δi,k2[+4ρks^k∣kı^k∣kΣs,kΣi,k]−s^k+1∣kı^k+1∣k.

## APPENDIX B

### ADAPTIVE ESTIMATION OF THE INFECTION RATE SLOPE

Let us assume that the infection rate in the time interval [*k* – *L*, *k*] is linearly changing according to the following model

(41) $$\beta_{k-\ell +1}=\beta_{k-\ell }+{\dot{\beta }}_{k}\Delta_{t}+\omega_{k,\ell }\;,$$

for ℓ ∈ {1, … , *L*}, where $\omega_{k,l}\;∼\;N(0,\sigma_{\omega }^{2})$ and ${\dot{\beta }}_{k}$ is a constant slope. An estimate of the slope can be obtained through the simple linear regression estimator [44] using the

**Table:**

Algorithm 1 Epidemic Estimation with Stochastic SIR Model
Input:k,z1:k=[z1T,…,zkT],N,D,β¯0,σβ2,γ¯0,σγ2,{x¯0(n),C¯0(n)}n=1NOutpu:{P(xk′∣θk′,z1:k′),P(θk′∣z1:k′),s^k′E,ı^k′E,β^k′E,γ^k′E}k′=1kINITIALIZATION1:k′←12:forθk′=[βk′,γk′]T∈Ddo3:P(θk′∣z1:k′−1)←N(βk′;β¯0,σβ2)N(γk′;γ¯0,σγ2)4:forn=1toNdo5:wk′∣k′−1(n,θk′)←N−16:x^k′∣k′−1(n,θk′)←x¯0(n)7:C^k′∣k′−1(n,θk′)←C¯0(n)8:endfor9:endfor10:fork′=1tokdoUPDATE11:forθk′∈Ddo12:forn=1toNdo13:αk′∣k′−1(n,θk′)←N(zk′;h1(x^k′∣k′−1(n,θk′))),(R(x^k′∣k′−1(n,θk′))+HC^k′∣k′−1(n,θk′)HT)14:wk′∣k′(n,θk′)←αk′∣k′−1(n,θk′)wk′∣k′−1(n,θk′)∑n′=1Nαk′∣k′−1(n′,θk′)wk′∣k′−1(n′,θk′)15:x^k′∣k′(n,θk′)←x^k′∣k′−1(n,θk′)+Kk′∣k′−1(n,θk′)[zk′−h1(x^k′∣k′−1(n,θk′))]16:C^k′∣k′(n,θk′)←C^k′∣k′−1(n,θk′)−Kk′∣k′−1(n,θk′)HC^k′∣k′−1(n,θk′)17:endfor18:P(xk′∣θk′,z1:k′)←∑n=1Nwk′∣k′(n,θk′)N(xk′;x^k′∣k′(n,θk′),C^k′∣k′(n,θk′))19:P(θk′∣z1:k′)←P(θk′∣z1:k′−1)∑n=1Nαk′∣k′−1(n,θk′)wk′∣k′−1(n,θk′)∑θk′′∈DP(θk′′∣z1:k′−1)∑n′=1Nαk′∣k′−1(n′,θk′′)wk′∣k′−1(n′,θk′′)20:endfor21:Computes^k′E,ı^k′E,β^k′E,andγ^k′Eaccording to (37)−(40)PREDICTION22:forθk′+1∈Ddo23:P(θk′+1∣z1:k′)←∑θk′∈DP(θk′+1∣θk′)P(θk′∣z1:k′)24:n′←125:forθk′∈Ddo26:forn=1toNdo27:wk′+1∣k′(n′,θk′+1)=wk′+1∣k′(n,θk′+1,θk′)←P(θk′+1,∣θk′)P(θk′∣z1:k′)P(θk′+1∣z1:k′)wk′∣k′(n,θk′)28:x^k′+1∣k′(n′,θk′+1)←x^k′+1∣k′(n,θk′)(via moment matching)29:C^k′+1∣k′(n′,θk′+1)←C^k′+1∣k′(n,θk′)(via moment matching)30:n′←n′+131:endfor32:endfor33:endforPRUNING34:forθk′+1∈Ddo35:Sort in descending order theN×Dmixture components accordingto the weights{wk′+1∣k′(n,θk′+1)}n=1N×D,and retain the firstNelements36:forn=1toNdo37:wk′+1∣k′(n,θk′+1)←wk′+1∣k′(n,θk′+1)∑n′=1Nwk′+1∣k′(n′,θk′+1)38:endfor39:endfor40:endfor

*L* + 1 most recent MMSE estimates of the infection rate, that is,

$${\hat{\dot{\beta }}}_{L,k}=\frac{\sum_{\ell =0}^{L}\left(\ell -L∕2\right)\left({\hat{\beta }}_{k-L+\ell }^{E}-(L+1{)}^{-1}\sum_{\ell^{\prime }=0}^{L}{\hat{\beta }}_{k-\ell^{\prime }}^{E}\right)}{\Delta_{t}\sum_{\ell =0}^{L}{\left(\ell -L∕2\right)}^{2}}.$$

The estimator ${\hat{\dot{\beta }}}_{L,k}$ is unbiased, and its variance is

$${\dot{\sigma }}_{L,k}^{2}=\frac{{\hat{\sigma }}_{\omega ,L}^{2}}{\Delta_{t}^{2}\sum_{\ell =0}^{L}{\left(\ell -L∕2\right)}^{2}},$$

where ${\hat{\sigma }}_{\omega ,L}^{2}$ is an estimate of $\sigma_{\omega }^{2}$, i.e., the variance of the noise term *ω*_*k*,ℓ_, given by

$${\hat{\sigma }}_{\omega ,L}^{2}=\frac{\sum_{\ell =1}^{L}{\left({\hat{\beta }}_{k-\ell +1}^{E}-{\hat{\beta }}_{k-\ell }^{E}-\Delta_{t}{\hat{\dot{\beta }}}_{L,k}\right)}^{2}}{L-1}.$$

In practice, the slope ${\dot{\beta }}_{k}$ is piecewise constant, that is, constant over subintervals of the entire interval [*k* – *L*, *k*]. Therefore, we aim to find the value *L*^★^, *L*_min_ ⩽ *L*^★^ ⩽ *L*_max_, — hence, the subinterval [*k* – *L*^★^, *k*] — such that the linear model with constant slope in (41) is a valid assumption for the time evolution of the infection rate. Let $\bar{L}$ be the candidate value for *L*^★^, and let us define the auxiliary variable $y_{\bar{L},k}$ as

(42) $$y_{\bar{L},k}≜\frac{{\left({\hat{\beta }}_{k}^{E}-{\hat{\beta }}_{k-1}^{E}-{\hat{\dot{\beta }}}_{\bar{L},k}\Delta_{t}\right)}^{2}}{{\hat{\sigma }}_{\omega ,L}^{2}}.$$

It is easy to verify that, if the linear model (41) is valid and ${\hat{\dot{\beta }}}_{\bar{L},k}$ represents an accurate estimate of the constant slope over the interval [$k-\bar{L}$, *k*], then $y_{\bar{L},k}$ is a central chi-square distributed variable with one degree of freedom, i.e., $y_{\bar{L},k}\;∼\;\chi_{1}^{2}(0)$; otherwise, $y_{\bar{L},k}$ is a non-central chi- square distributed variable with one degree of freedom and non-centrality parameter *ν* > 0, i.e., $y_{\bar{L},k}\;∼\;\chi_{1}^{2}(v)$. The problem of finding *L*^★^ can therefore be recast as a hypothesis test: the null hypothesis (*H*_0_) states that the linear assumption is valid and ${\hat{\dot{\beta }}}_{\bar{L},k}$ is a good estimate of the constant slope; the alternate hypothesis (*H*_1_) states that the linear hypothesis with constant slope is invalid. The hypothesis test can be formally written as

{H0:yL¯,k∼χ12(0),H1:yL¯,k∼χ12(v),}

and the decision rule is

$$y_{\bar{L},k}\overset{H_{0}}{\underset{H_{1}}{≶}}T_{H}.$$

The threshold *T*_*H*_ is obtained as

$$T_{H}=F_{\chi_{1}^{2}(0)}^{-1}(1-P_{\text{fa}}),$$

where *P*_fa_ is the probability to reject hypothesis *H*_0_ when it is true and $F_{\chi_{1}^{2}(0)}^{-1}(\cdot )$ is the inverse cumulative density function of a central chi-square variable with one degree of freedom. In order to select *L*^★^, we employ the following iterative procedure: if with $\bar{L}$ hypothesis *H*_0_ is accepted, and with $\bar{L}-1$ hypothesis *H*_0_ is rejected, then $L^{⋆}\;=\;\bar{L}$; otherwise, $\bar{L}$ is decreased and the test is repeated. This iterative procedure starts with $\bar{L}=L_{\max}$. Note that, alternatively, the threshold could be obtained by fixing the probability to accept hypothesis *H*_0_ when it is false; in such a case, the non-centrality parameter *ν* is approximated with the numerator of (42).

**Table:**

Algorithm 2 Epidemic Forecasting with Stochastic SIR Model
$$\begin{array}{cc} \; & \;\mathrm{Input}:\;k,P(x_{k}|z_{1:k}),P(\theta_{k}|z_{1:k}),J,L,K,P,\{{\hat{\beta }}_{k^{\prime }}^{E}{\}}_{k^{\prime }=k-L}^{k}\\ \; & \;\mathrm{Output}:\{{\hat{s}}_{k^{\prime }}^{F},{\hat{ı}}_{k^{\prime }}^{F},{\hat{\beta }}_{k^{\prime }}^{F},{\hat{\gamma }}_{k^{\prime }}^{F}{\}}_{k^{\prime }=k+1}^{k+K}\\ \; & \;\;\;\;\;\text{INITIALIZATION}\\ \; & \;\;1:\;\text{Draw}\;J\;\text{samples}\;{\tilde{x}}_{k}^{\left(j\right)}=[{\tilde{s}}_{k}^{\left(j\right)},{\tilde{ı}}_{k}^{\left(j\right)}{]}^{T}\;\text{from}\;P(x_{k}|z_{1:k})\\ \; & \;\;2:\;\text{Draw}\;J\;\text{samples}\;{\tilde{\theta }}_{k}^{\left(j\right)}=[{\tilde{\beta }}_{k}^{\left(j\right)},{\tilde{\gamma }}_{k}^{\left(j\right)}{]}^{T}\;\text{from}\;P(\theta_{k}|z_{1:k})\\ \; & \;\;3:\;{\hat{\dot{\beta }}}_{L,k}\leftarrow \frac{\sum_{\ell =0}^{L}(\ell -L∕2)({\hat{\beta }}_{k-L+\ell }^{E}-(L+1{)}^{-1}\sum_{\ell^{\prime }=0}^{L}{\hat{\beta }}_{k-\ell^{\prime }}^{E})}{\Delta_{t}\sum_{\ell =0}^{L}(\ell -L∕2{)}^{2}}\\ \; & \;\;4:\;{\hat{\sigma }}_{\omega ,L}^{2}\leftarrow \frac{\sum_{\ell =1}^{L}{\left({\hat{\beta }}_{k-\ell +1}^{E}-{\hat{\beta }}_{k-\ell }^{E}-\Delta_{t}{\hat{\dot{\beta }}}_{L,k}\right)}^{2}}{L-1}\\ \; & \;\;5:\;{\dot{\sigma }}_{L,k}^{2}\leftarrow \frac{{\hat{\sigma }}_{\omega ,L}^{2}}{\Delta_{t}^{2}\sum_{\ell =0}^{L}{\left(\ell -L∕2\right)}^{2}}\\ \; & \;\;\;\;\;\text{FORECASTING}\\ \; & \;\;6:\;\mathrm{for}\;j=1\;\mathrm{to}\;J\;\mathrm{do}\\ \; & \;\;7:\;\;\;\;\mathrm{for}\;k^{\prime }=k+1\;\mathrm{to}\;k+K\;\mathrm{do}\\ \; & \;\;\;\;\;\;\;\;\;\;\text{STATE EVOLUTION}\\ \; & \;\;8:\;\;\;\;\;\;\;\text{Draw}\;\lambda =[\lambda_{1},\lambda_{2}{]}^{T}∼N(0,I_{2}\Delta_{t})\\ \; & \;\;9:\;\;\;\;\;\;\;\sigma_{1,k^{\prime }-1}^{\left(j\right)}\leftarrow \sqrt{P^{-1}{\tilde{\beta }}_{k^{\prime }-1}^{\left(j\right)}{\tilde{s}}_{k^{\prime }-1}^{\left(j\right)}{\tilde{ı}}_{k^{\prime }-1}^{\left(j\right)}}\\ \; & \;10:\;\;\;\;\;\;\;\sigma_{2,k^{\prime }-1}^{\left(j\right)}\leftarrow \sqrt{P^{-1}{\tilde{\gamma }}_{k^{\prime }-1}^{\left(j\right)}{\tilde{ı}}_{k^{\prime }-1}^{\left(j\right)}}\\ \; & \;11:\;\;\;\;\;\;\;{\tilde{s}}_{k^{\prime }}^{\left(j\right)}\leftarrow {\tilde{s}}_{k^{\prime }-1}^{\left(j\right)}-{\tilde{\beta }}_{k^{\prime }-1}^{\left(j\right)}{\tilde{s}}_{k^{\prime }-1}^{\left(j\right)}{\tilde{ı}}_{k^{\prime }-1}^{\left(j\right)}\Delta_{t}+\sigma_{1,k^{\prime }-1}^{\left(j\right)}\lambda_{1}\\ \; & \;12:\;\;\;\;\;\;\;{\tilde{ı}}_{k^{\prime }}^{\left(j\right)}\leftarrow {\tilde{ı}}_{k^{\prime }-1}^{\left(j\right)}+{\tilde{\beta }}_{k^{\prime }-1}^{\left(j\right)}{\tilde{s}}_{k^{\prime }-1}^{\left(j\right)}{\tilde{ı}}_{k^{\prime }-1}^{\left(j\right)}\Delta_{t}\\ \; & \;\;\;\;\;\;\;\;\;\;\;\;\;\;\;\;\;\;\;-{\tilde{\gamma }}_{k^{\prime }-1}^{\left(j\right)}{\tilde{ı}}_{k^{\prime }-1}^{\left(j\right)}\Delta_{t}-\sigma_{1,k^{\prime }-1}^{\left(j\right)}\lambda_{1}+\sigma_{2,k^{\prime }-1}^{\left(j\right)}\lambda_{2}\\ \; & \;\;\;\;\;\;\;\;\;\;\text{PARAMETERS EVOLUTION}\\ \; & \;13:\;\;\;\;\;\;\;\text{Draw}\;\eta ∼N(0,{\dot{\sigma }}_{L,k}^{2})\\ \; & \;14:\;\;\;\;\;\;\;{\tilde{\beta }}_{k^{\prime }}^{\left(j\right)}\leftarrow {\tilde{\beta }}_{k^{\prime }-1}^{\left(j\right)}+{\hat{\dot{\beta }}}_{L,k}\Delta_{t}+\eta \\ \; & \;15:\;\;\;\;\;\;\;{\tilde{\gamma }}_{k^{\prime }}^{\left(j\right)}\leftarrow {\tilde{\gamma }}_{k^{\prime }-1}^{\left(j\right)}\\ \; & \;\;\;\;\;\;\;\;\;\;\;\text{ENSEMBLE MEAN}\\ \; & \;16:\;\;\;\;\;\;\;{\hat{s}}_{k^{\prime }}^{F}\leftarrow J^{-1}\sum_{j=1}^{J}{\tilde{s}}_{k^{\prime }}^{\left(j\right)}\\ \; & \;17:\;\;\;\;\;\;\;{\hat{ı}}_{k^{\prime }}^{F}\leftarrow J^{-1}\sum_{j=1}^{J}{\tilde{ı}}_{k^{\prime }}^{\left(j\right)}\\ \; & \;18:\;\;\;\;\;\;\;{\hat{\beta }}_{k^{\prime }}^{F}\leftarrow J^{-1}\sum_{j=1}^{J}{\tilde{\beta }}_{k^{\prime }}^{\left(j\right)}\\ \; & \;19:\;\;\;\;\;\;\;{\hat{\gamma }}_{k^{\prime }}^{F}\leftarrow J^{-1}\sum_{j=1}^{J}{\tilde{\gamma }}_{k^{\prime }}^{\left(j\right)}\\ \; & \;20:\;\;\;\;\mathrm{end}\;\mathrm{for}\\ \; & \;21:\;\mathrm{end}\;\mathrm{for} \end{array}$$
